# Nurses, non-nurse healthcare providers, and clients’ perspectives, encounters, and choices of nursing gender in Tanzania: a qualitative descriptive study

**DOI:** 10.1186/s12912-024-02027-3

**Published:** 2024-05-27

**Authors:** Racheal Mukoya Masibo, Stephen M. Kibusi, Golden M. Masika

**Affiliations:** School of Nursing and Public Health, The University of Dodoma, Dodoma, Tanzania

**Keywords:** Perspective, Nursing, Gender, Preferences, choice

## Abstract

**Background:**

A growing share of male nurses in the nursing profession in Tanzania has changed the trend of diversity of nursing gender. This might have created a divergent perspective within the communities. Therefore, the current study aimed to explore the perspective, encounters, and choices of nursing gender among licensed nurses, non-nurse healthcare providers, and clients in Tanzania.

**Methods:**

The study employed a qualitative descriptive design. The data were collected between August 2022 to January 2023 by the principal investigator and one research assistant. Twelve Focus Group Discussions (FGDs) were carried out in four different hospitals in Dar es Salaam comprised of total participants (*n* = 59). The participants were nurses, clients, and non-nurse healthcare providers. The data was collected through an interview guide developed by the principal investigator and validated by nurse experts. The data was analyzed using qualitative content analysis to generate themes and subthemes.

**Results:**

Eight themes and twenty-seven subthemes emerged from the study. The following are themes; ① Variations of male and female nurses in communication ② Differences of male and female nurses in carrying out leadership roles ③ Divergent clinical qualities and outcomes across nursing gender ④ Positive value of male nurses in clinical facilities from colleagues and patients ⑤ Different cooperation of male and female nurses at the clinical settings ⑥ Mixed perspective towards clinical competencies across nursing gender ⑦ Perspective towards gender diversity in nursing ⑧ Preferences of nurse’s gender, reasons, and opinion towards gender preferences.

**Conclusion:**

Male nurses and female nurses differ in how they communicate, execute leadership roles, and clinical qualities. However, their variations don’t mean one gender is underrated than the other, but every gender has unique communication styles, leadership styles, and clinical qualities that both lead to effective outcomes. Diversity in nursing gender is very important and should be strategized. Since preferences of nursing gender seems to enhance somebody’s freedom and creates an environment where a person can discuss sensitive issues, nursing bodies and healthcare stakeholders might initiate a discussion about approaches to promote the implementation of nursing preference and perform the feasibility studies.

**Supplementary Information:**

The online version contains supplementary material available at 10.1186/s12912-024-02027-3.

## Introduction

Nursing is one of the most gendered professions globally, dominated by females and with little representation of males [1]. The data by the World Health Organization from 104 countries conform to the statement that men are underrepresented in nursing as follows; Africa (65%=Female nurses and 35%=Male nurses) [2], America (86%=Female nurses and 14%=Male nurses), Eastern Mediterranean (79%=Female nurses and 21%=Male nurses), Europe (84%=Female nurses and 16%=Male nurses), South-East Asia (79%=Female nurses and 21%=Male nurses) and Western Pacific (81%=Female nurses and 19%=Male nurses) [3]. Generally, male nurses make up 11% of the nursing workforce worldwide, while female nurses comprise 89% of the nursing workforce [4]. Following the disparity in nursing gender, the recent campaigns to encourage and attract more men to nursing are promising evidenced high the influx of men choosing nursing as a career [5]. The influx has slightly increased the trend of nursing gender, considering the number of employed male nurses in the United States has increased from 11.1% in 2019 to 13.3% in 2021, while the enrollment of male nurses in the program Doctor of Nursing Practice (DNP) has increased from 13.4% in 2018 to 14.8% in 2022 [2].

It is hypothetically assumed that having gender diversity in nursing is associated with the acceleration of professional development, a force for public policy change, and improving patient care by providing an opportunity to choose the gender of their preference [6]. Consistently gender theories put forth that gender diversity is good, for instance, Symbolic Interaction Theory in Gender sees gender diversity as a factor that allows us to form a relationship in daily life [7], while another theory by Bonnie, proposed that looking at masculinity and femininity assets of mutually created characteristics shape the lives [8]. Even though the benefits of gender diversity are reported hypothetically, there is a limited reported actual benefit in clinical settings. The study ought to explore the contribution of gender trends in the clinical areas.

The background of nursing shows that the profession’s image has always been determined by the public perspectives, whereby these perspectives have the power to affect the users of health services, the nurses’ performance, health policy, and even the choice to become a nurse [9]. Therefore, the changing trend of gender diversity of nurses triggers public perspective about the impact of diversity in nursing care, collaboration aspects, image of the profession, retention of male nurses, and preferences of nursing gender by patients [10]. Even though some studies have been previously conducted to discover the public perspective on nursing gender [11, 12], there are still conflicting results as some reported positive perspectives and other negative perspectives and most of the studies targeted nursing students and patients while abandoned licensed nurses and non-healthcare providers. The current study involved licensed nurses, non-healthcare providers, and clients. Nursing gender disparity evolved since the origin of the nursing profession when women were considered fitting to care for clients because of their experiences in traditional caring roles like raising children, feeding and caring for members of the household, and organizing and maintaining the home, unlike men who were viewed to be in a position of engaging in productive roles [13]. The gender disparity was further exacerbated by Florence Nightingale in the 19th century, where she envisioned nursing as the most suitable profession for women because it was an extension of mothering [14]. In this aspect of gender disparity, every woman was thought to qualify as a nurse and men were refused to be registered as nurses [15–17].

However, with the 1970 campaign for gender balance, more men were recruited into nursing by downsizing the ‘feminine’ attributes in nursing and refocusing on the governance and scientific aspects [15]. Since 1970 to date the number of male nurses has tripled [18]. Studies have suggested the presence of competitive salaries, professional stability, shortage of nurses, leadership opportunities, ageing population, ageing workforce, burnout, vibrant job employment competition, and career development opportunities to be the reasons men are joining the nursing profession [19, 20]. Even though there is adequate documentation about changing gender trends in nursing, previous studies have not captured the impact of the changing gender trends on nursing professional practice.

In Tanzania, in 2013, the disparity of nursing gender has been high, with female nurses (86%) and male nurses (14%) [21]. In 2015, the proportion of gender distribution of nurses changed with female nurses (70%) and male nurses (30%) [22]. The raised gender diversity is perpetuated by the unemployment rate, job stability, career flexibility, growth of the nursing field, rising income, changing of nursing entry qualifications, and presence of a variety of specialities in nursing [23, 24]. Even though no published statistics on the changing trend of gender in nursing, the grey literature from the registry of Tanzania Nursing and midwifery council indicates a notable increase in male nurses at the bachelor’s level from 2015 to 2022 as follows; 2015 (540 = Female nurse and 167 = Male nurses), 2016 (565 = Female nurses and 205 = Male nurses), 2017 (501 = Female nurses and 221 = Male nurses), 2018 (508 = Female nurses and 242 = Male nurses), 2019 (410 = Female nurses and 272 = Male nurses), 2020 (362 = Female nurses and 310 = Male nurses), 2021 (311 = Female nurses and 337 = Male nurses), and 2022 (345 = Female nurses and 352 = Male nurses). This conforms to unpublished statistics and reports by Tanzania Nursing and Midwifery Council (TNMC), Tanzania Commission for Universities (TCU), and current qualitative findings of 15 experienced Tanzania nurses, which uncovered the changing gender trends in the enrollment of male student nurses pursuing nursing careers.

Since the public perspective toward nursing has been an issue in Tanzania throughout the transition stages of nursing development [25–28], the public perspective towards changing trends of nursing gender cannot be avoided.

The evolving gender trends in the nursing workforce call for an urgent investigation of perspectives towards the changing trend and its impact on the nursing professional practice. Moreover, the limited studies on the perspective of nursing gender increase the need to conduct this study. Therefore, the current study aimed to explore the perspectives, encounters, and choices of nursing gender among licensed nurses, non-nurse healthcare providers, and clients in Tanzania. The study objectives are; (1) to explore nurses, non-nurse healthcare providers, and clients’ perspectives towards nursing gender in Tanzania, (2) to explore the opinions of nurses, non-nurse healthcare providers, and clients towards nursing gender and (3) to describe the nursing gender preference among nurses, non-nurse healthcare providers, and clients.

## Methods

### Study designs

It is a qualitative descriptive design that explores the perspectives, euncounter and choices of nursing gender among nurses, non-nurse healthcare providers, and clients in Tanzania. The design was appropriate as it can generate data about ‘who, what, and where of events and is deemed important because it provides straightforward descriptions of experiences and perspectives [29].

### Study setting and population

The study was conducted in Tanzania specifically in Dar es Salaam, because of the increased gender diversity workforce in different healthcare facilities in the urban of Dar es Salaam. There is evidence in Tanzania showing a link between gender workforce and geographically located facilities, whereas urban facilities are likely to have diverse genders, unlike rural facilities with fewer female professionals [21]. Moreover, due to the accessibility of basic social services and desirable infrastructural facilities, most of the qualified professionals 69% are concentrated in urban areas [30].

Therefore, the study involved four hospitals located in Dar es Salaam, named as Hospital 1, Hospital 2, Hospital 3, and Hospital 4. In detail, Hospital 1 has the bed capacity 1,500, with 2,000 outpatients attending per day, and with 2,800 employees. Meanwhile, Hospital 2 has 362 beds and provides additional daily care for 800-1,200 outpatients, with an estimated total of employee 435. Furthermore, Hospital 3 in 2018 had a bed capacity of 230, serving 1.4 million population, while Hospital 4 serving a population of more 2.2 million people, has a bed capacity of 254, with a bed occupancy of 317, and attends 1,500 up to 1,800 patients per day. The study involved licensed nurses working at healthcare facilities, non-nurse healthcare providers (physicians, laboratory technicians, and pharmacists), and clients who attended healthcare facilities to seek medical attention at the time of the study.

### Inclusion and exclusion criteria

Nurses, and non-nurse healthcare providers who are licensed and currently working at the healthcare facilities were included in the study, but those unwilling to participate and absent during data collection were excluded. Meanwhile, clients who were able to speak Swahili native language or English were recruited, but those who did not complete informed consent and with severe illnesses to impaired their responses were excluded from the study.

### Sample size and sampling procedures

The sample size was determined based on data saturation. When no new matters/issues were emerging from Focus Group Discussions (FGDs), the data were considered saturated. Enough participation from each member in each focus group session and exploring exhaustively in each focus group was performed to ensure no new ideas were coming out. Therefore, there were four FGDs for nurses, four FGDs for non-nurse healthcare providers, and four FGDs for clients. Regarding the sampling procedure, a purposive method was used to recruit participants.

### Data collection procedures and tools

The data were collected between August 2022 to January 2023 by the principal investigator and one research assistant. The assistant was a licensed registered nurse who worked as ward-in-charge at a hospital in Dar es Salaam. Twelve Focus Group Discussions (FGDs) were carried out in the four hospitals with three FGDs in each hospital which comprised of nurses, non-nurse healthcare providers, and clients respectively. Each FGD comprised 5–6 participants who shared their perspectives, encounters, and choices about nursing gender in Tanzania. This number of participants per single FGD is based on the recommended minimum number of five participants needed to conduct a single Focus [31].

To formulate the FGDs of nurses at different facilities, the directors of nursing services were physically met and asked for a list of nurses with the required criteria. The selected nurses were contacted physically or through a mobile phone, and were informed about the study, completed consent, and scheduled the date and time for interviews. For non-nurse healthcare providers (NN-HCPs), the directors of medical services, directors of pharmacy, and directors of laboratories at different facilities were also asked to provide a list of potential participants with the required criteria to participate in the study. After getting a list, the proposed participants were contacted and asked to participate in the study. The FGDs were formulated and all participants were informed of the time for an interview. For all FGDs across three populations, the balance of gender was deliberately ensured to promote diversity.

### Reflexivity and bracketing

Team members were mixed of female and male academicians, with diverse backgrounds in nursing care, the nursing workforce, nursing management, public health, and global health. One team member (RMM) was an early career researcher (2 years experience) and Two of the team members (SMK & GMM) had more than 10 years of experience in nursing qualitative research. This experience was valuable in the design of the study, recruitment of participants and data collection. During the analysis we used an inductive approach which was grounded in the data, to minimise the impact of the researchers on the findings. All authors had demographics that would have influenced the findings. However, researchers self-consciously critique, appraise, and evaluate how their subjectivity and context influence the research processes helped to mitigate the effect. Moreover, methodological cohesion, working inductively, and acquiring adequate and appropriate samples further prevented the researcher’s characteristics from influencing the results [32].

### Data collection tools

The same interview guide for different populations was used to guide the interview sessions. The English interview guide was developed by a principal investigator based on literature and study objectives. The validation was performed by an expert in qualitative studies, who reviewed the interview guide and provided comments for revision. The final English interview guide contained three main questions (1) What is your perspective of nursing gender in Tanzania? (2) What is your opinion about nursing gender in Tanzania? (3) What can you say about your preference for nursing gender when you are seeking medical attention?. The final English version was translated into Swahili native language and interview sessions were conducted in Swahili native language, for convenience of participatns. The probing questions during interview sessions were based on the information from participants, such as “Can you explain more about what you have just said?” “Can you give an example of what you have mentioned?” “Do you think there are still other issues you want to speak about?” “Has anything important been left out or forgotten that you would like to share?” Meanwhile, the audio recorder was used to capture the conversations that conveniently helped during data analysis. Refer to supplementary Data 1.

### Data analysis

The collected data were transcribed verbatim into text by the principal investigator. The transcripts were translated forward and backward by a linguist, working as an English lecturer at St. John’s University of Tanzania. A principal investigator and one co-author coded the data independently. Every coded data was supposed to have an agreement between two coders, but a third co-author was consulted upon lack of agreement between two coders. Qualitative Content Analysis was used to analyze the data through the following steps; familiarizing oneself with the data, dividing up the text into meaning units and condensing meaning units, formulating codes, and developing categories and themes [33]. The coding data can be referred to Supplementary Data 2–4.

### Ethics approval and consent to participate

The study ethical clearance letter was obtained from the University of Dodoma Institution Research Review Committee (IRREC), with reference number: MA 84/261/02. The permission to conduct the study in four hospitals was obtained from the Regional Administrative Secretary (RAS). The written and verbal informed consent was completed by each participant before participating in the study. None of the participants was under 16 years of age, and therefore no guardian completed the informed consent on behalf of the participants. Participants had the freedom to participate voluntarily and withdraw from the study at any time they felt so. Since the study involved FGDs, full confidentiality could not be fully guaranteed [34], but permission to record and transcribe the data was obtained. Moreover, participants have explained procedures for how their recordings will be kept confidential, such as avoiding exposing participants’ identities, deleting the recordings and destroying transcripts six months after analysis, and securing devices containing recording files by passwords and encryption.

The researcher assistant had a greater chance of encountering conflicts of interest, since she is working in one of the studied hospitals. The conflict could have been raised when she was required to decide whether to defend the interest of her hospital or be led by study objective. The agreement with the research assistant before carrying out the study was the interview sessions should be guided by the interview guide and principles.

### Trustworthiness

The credibility which deals with how congruent are the findings with reality was ensured through triangulation [35] and member checks. Two type of trianglulation that helped to make sure that the research findings are robust, rich, comprehensive, and well-developed ae; (i) triangulation of sources (collecting data from multiple sources of three populations) and (ii) analyst triangulation (having multiple analysists of qualitative data). Furthermore, the credibility was achieved through member checks, where the data, interpretations, and conclusions about nursing gender perspectives, encounters and choices were shared with the participants to enable them clarify what their intentions were, correct errors, and provide additional information if necessary. Detailed information was provided within the methodological section, including study setting, data saturation, sampling procedure, techniques of data collection, and tool development and validation to achieve transferability. Meanwhile, the dependability was ensured by involving multiple people in reading transcripts for face validity. Bracketing, reflexivity, and clear coding were used for confirmability.

## Results

### Participants’ characteristics

The data were collected at four different hospitals, with a total of 12 Focus Group Discussions (FGDs). The total participants in the study were fifty-nine, distributed as 20 nurses, 16 non-nurse healthcare providers, and 23 clients.

Since FGD was an approach for data collection, a single FGD for each population was conducted at each hospital. The data collected from nurses in each hospital were; at Hospital 1: the FGD comprised five nurses (3 = female and 2 = males), with work experience ranging from 7 to 10 years. The FGD at Hospital 2 had six nurses (4 = males and 2 = females) who had worked for 2 to 8 years. Meanwhile, the FGD at Hospital 3 had five nurses (2 = males and 3 = females), having the working experience ranging from 5 to above 10 years. The FGD at Hospital 4 involved five nurses (3 = females and 2 = males) who had worked between 6 and beyond 10 years.

The data were also collected from non-nurse healthcare providers (NN-HCPs) who were medical doctors, laboratory technicians, and pharmacists. Four FGDs were carried out at four hospitals. At Hospital 1, a total of 4 participants were involved in FGD whereby there were two medical doctors (1 = male and 1 = female), one pharmacist (male), and one lab technician (male), with working experience ranging from 5 and over 15 years. At hospital 2, the FGD comprised four participants, two physicians (1 = male and 1 = female), one pharmacist (female), and one laboratory technician (female), with working experience of 7 years. Moreover, Hospital 3 had four participants comprising of, two medical doctors (1 = male and 1 = female), one pharmacist (female), and one medical laboratory scientist (male) who had worked in their careers between 5 and 7 years. At Hospital 4, four participants comprising of, two medical doctors (1 = male and 1 = female), one pharmacist (female), and one medical laboratory scientist (male) having working experience ranging from 10 to 22 years participated in the FGD.

Additionally, the data from clients were collected from four hospitals, with a total of four FGD and a single FGD per hospital. A total of 23 clients participated across all FGDs (14 = Female & 9 = Male). At Hospital 1 the FGD had six participants (4 = Female and 2 = Male) and at Hospital 2 the FGD had six participants (3 = Female and 3 = Male). Additionally, at Hospital 3 the FGD had six participants (3 = Female and 3 = Male), while at Hospital 4 the FGD had five participants (4 = Female and 1 = Male). Refer to Table 1.

**Table 1:** Participants’ characteristics

	Facilities	Interviewee in FGD	Sex	Professional	Experience
**Nurses**	HOSPITAL 1	P1	Female	Nurse	10 years
		P2	Male	Nurse	-
		P3	Female	Nurse	7 years
		P4	Male	Nurse	-
		P5	Female	Nurse	9 years
	HOSPITAL 2	P1	Male	Nurse	5 years
		P2	Male	Nurse	8 years
		P3	Male	Nurse	2 years
		P4	Male	Nurse	4 years
		P5	Female	Nurse	-
		P6	Female	Nurse	-
	HOSPITAL 3	P1	Male	Nurse	Over 10 years
		P2	Female	Nurse	8 years
		P3	Male	Nurse	5 years
		P4	Female	Nurse	-
		P5	Female	Nurse	-
	HOSPITAL 4	P1	Male	Nurse	6 years
		P2	Female	Nurse	5 years
		P3	Female	Nurse	Over 10 years
		P4	Female	Nurse	-
		P5	Male	Nurse	-
**Non-Nurse Healthcare providers**	HOSPITAL 1	P1	Male	Medical doctor	Over 15 years
		P2	Male	Pharmacist	Over 8 years
		P3	Male	Lab technician	7 years
		P4	Female	Medical doctor	Over 5 years
	HOSPITAL 2	P1	Male	Medicine (Physician)	-
		P2	Female	Pharmacist	-
		P3	Female	Lab technician	-
		P4	Female	Medicine (Physician)	-
	HOSPITAL 3	P1	Female	Pharmacist	7 years
		P2	Male	Medical laboratory scientist	-
		P3	Female	Medical doctor	6 years
		P4	Male	Medical doctor	5 years
	HOSPITAL 4	P1	Male	Senior medical laboratory scientist	22 years
		P2	Male	Medical doctor	15 years
		P3	Female	Pharmacist	-
		P4	Female	-	10 years
**Clients**	HOSPITAL 1	P1	Female	-	-
		P2	Male	-	-
		P3	Female	-	-
		P4	Female	-	-
		P5	Male	-	-
		P6	Female	-	-
	HOSPITAL 2	P1	Female	-	-
		P2	Female	-	-
		P3	Female	-	-
		P4	Male	-	-
		P5	Male	-	-
		P6	Male	-	-
	HOSPITAL 3	P1	Male	-	-
		P2	Female	-	-
		P3	Female	-	-
		P4	Male	-	-
		P5	Male	-	-
		P6	Female	-	-
	HOSPITAL 4	P1	Male	-	-
		P2	Female	-	-
		P3	Female	-	-
		P4	Female	-	-
		P5	Female	-	-

### Themes

Through content analysis, ten themes and thirty-three subthemes emerged from the study. The following are the themes; ① Variations of male and female nurses in communication ② Differences of male and female nurses in carrying out leadership roles ③ Divergent clinical qualities and outcomes across nursing gender ④ Positive value of male nurses in clinical facilities from colleagues and patients ⑤ Different cooperation of male and female nurses at the clinical settings ⑥ Mixed perceptions towards clinical competencies across nursing gender ⑦ Perspective towards gender diversity in nursing ⑧ Preferences of nurse’s gender, reasons, and opinion towards gender preferences. Refer to Table 2.

**Table 2:** Summary of themes and subthemes of the current study

S/NO	Subthemes	Themes
1	*The existing variations in communication between male and female nurses*	① Variations of male and female nurses in communication and leadership
2	*Areas of difference in communication between male and female nurses*	
3	*Factors for communication differences between male and female nurses*	
1	*The existing variation in leadership between male and female nurses*	② Differences of male and female nurses in carrying out leadership roles
2	*Different leadership style used by male and female nurses*	
1	*Both male and female nurses’ positive qualities in clinical practice*	③ Divergent clinical qualities and outcomes across nursing gender
2	*Male nurses’ positive qualities and impact in clinical practice*	
3	*Male nurses’ negative qualities and effect on clinical practice*	
4	*Female nurses’ positive qualities and outcomes in clinical practice*	
5	*Female nurses’ negative qualities and repercussions in clinical practice*	
1	*Value of male nurses by colleague*	④ Positive value of male nurses in clinical facilities from colleagues and patients
2	*Value of male nurses from patients*	
1	*Good interaction of male and female nurses*	⑤ Different cooperation of male and female nurses at the clinical settings
2	*Poor interaction of male and female nurses in clinical practice*	
1	*Negative perspective towards male nurses’ clinical competency*	⑥ Mixed perceptions towards clinical competencies across nursing gender
2	*Negative perspective towards female nurses’ clinical competency*	
1	*The need for diversity in nursing*	⑦ Perspective towards gender diversity in nursing
2	*The demand to address gender biases in nursing*	
3	*The relation between nursing gender and competence*	
4	*Distinct importance of nursing gender diversity to patients and nurses*	
1	*Having no gender preference for nurses*	⑧ Preferences of nurse’s gender, reasons, and opinion towards gender preferences
2	*Having preference of nurse of specific gender*	
3	*Having preference of nurse of same gender*	
4	*Having preference of nurse of opposite gender*	
5	*Reasons for gender preferences*	
6	*The challenge of meeting patients’ preferences through nurse’s gender diversity*	
7	*Different opinion about gender preferences*	

### Theme 1: variations of male and female nurses in communication

Both populations including nurses, non-nurse health care providers, and clients reported noticed differences in communication between female and male nurses. They differ in the communication approaches they use, and vary when conveying information to clients, and when discussing with other non-nurse healthcare providers. However, communication differences among female and male nurses are not influenced by their gender but rather by individual personalities, communication skills, and experience. The first theme had three sub-themes (Table 2), which are elaborated on below.

### Subtheme 1.1: the existing variations in communication between male and female nurses

It was mentioned by nurses in FGDs that female and male nurses differ in how they communicate

*“Communication styles can vary between male and female nurses” (FGD1-N)*.

Consistently, the clients confirm the existing variations of communication between male nurses and female nurses

*“I’m acknowledging that there might be variations in communication styles and approaches for male and female providers” (FGD4-C)*.

### Subtheme 1.2: areas of difference in communication between male and female nurses

Non-nurse HCPs in different FGDs revealed the presence of variation in how female and male nurses communicate, especially when conveying information to clients, when discussing with team members, and when discussing medication-related issues. When conveying information to clients, the difference is noticeable during normal conversation or when discussing medication.

*“I have observed differences in the communication styles between male and female nurses when conveying information about patient medications. Female nurses often tend to provide more detailed information, while male nurses tend to focus on the key points. Both are effective, but it’s interesting to note the variation” (FGD1-NN-HCP)*.

*“The variations in communication styles among male and female nurses that I might have noticed is when they are discussing medication-related matters. Female nurses give rather long and detailed approach when explaining medication instructions to clients, whereas male nurses might be a little brief” (FGD3-NN-HCP)*.

*“I have also observed that some and not all female nurses tend to be more empathetic and nurturing in their communication style, especially when discussing medication plans or side effects with clients. On the other hand, male nurses might focus more on factual information of which both of these approaches have their strengths, but these differences do impact patient interactions” (FGD4-NN-HCP)*.

NN-HCPs reported their experience, particularly the communication difference between male and female nurses based on the communication approach they use.

*“In my experience, there are occasional differences in communication styles between male and female healthcare providers. Female colleagues often bring a more empathetic and nurturing approach, which can be beneficial in certain patient interactions. On the other hand, male colleagues might sometimes adopt a more direct or assertive communication style although we also have some male colleagues who portray the stated empathetic approach” (FGD1-NN-HCP)*.

*“There have been instances where male nurses have been more concise and to the point in communicating medication orders, whereas female nurses might provide more contextual information about the patient’s condition” (FGD2-NN-HCP)*.

NN-HCPs also mentioned the existing differences in communication between male and female nurses when discussing with other healthcare providers.

*“In my experience, gender dynamics can influence communication styles within the healthcare team. For instance, I’ve noticed that there might be variations in how male and female colleagues express their opinions or share information during team discussions” (FGD1-NN-HCP)*.

*“I have noticed that sometimes, gender-related communication styles can impact how prescriptions or medication information are relayed and occasionally, there’s a difference in how male and female nurses approach discussing drug interactions or side effects with us” (FGD2-NN-HCP)*.

*“A male nurse might come in with a prescription and request for drugs and leave while a female nurse might ask some specific details as what type of food the patient should be told to eat and other basic stuff regarding the drugs. But also, not all female nurses do that because some male nurses inquire too though the percentage is small” (FGD2-NN-HCP)*.

### Subtheme 1.3: factors for communication differences between male and female nurses

There are factors beyond nursing gender influencing how female and male nurses communicate, especially individual personalities, communication skills, and experience.

*“It’s more about individual personalities and communication skills rather than a distinct difference based on gender” (FGD2-N)*.

*“It’s more about individual personalities and experiences shaping communication (FGD3-N)*.

*“Each individual brings their unique approach, influenced by personality and experience rather than being strictly gender-based” (FGD4-N)*.

### Theme 2: differences between male and female nurses in carrying out leadership roles

NN-HCPs revealed that nurse gender influences leadership capability. The variation is observed in the leadership style used by male and female nurses. Some use authoritative and others use democratic leadership style. The second theme had two sub-themes (Table 2), which are elaborated in detail below.

### Subtheme 2.1: the existing variation in leadership between male and female nurses

NN-HCPs have indicated that nurses of different genders when in positions of leadership, differ in executing leadership roles.

*“I have also noticed some slight variation in leadership styles between male and female nurses” (FGD3-NN-HCP).*

### Subtheme 2.2: different leadership styles used by male and female nurses

NN-HCPs mentioned that they have experienced seeing male nurses practicing an authoritative leadership style while female nurses assume a democratic leadership style. They added that the leadership style used by female nurses is good as it promotes staff involvement and enhances interaction.

*“Sometimes, female nurses exhibit more collaborative leadership, while male nurses might take a more authoritative approach. This can influence how tasks are delegated and how teamwork unfolds within the healthcare unit” (FGD3-NN-HCP).*

### Theme 3: divergent clinical qualities and outcomes across nursing gender

Most nurses, NN-HCPs, and clients in different FGDs reported variations of clinical qualities for male and female nurses. As it is with male nurses possess positive and negative qualities in clinical settings, and female nurses do. Yet, some participants in different FGDs did not mention separate qualities for male or female nurses but rather put forth the shared qualities among nursing genders. The third theme had five sub-themes (Table 2), which are elaborated on below.

### Subtheme 3.1: both male and female nurses’ positive qualities in clinical practice

Nurses in different FGDs indicated that male and female nurses have some common positive qualities in clinical areas, including being good at collaboration and exercising effective communication.

*“I’ have also had positive experiences of good collaboration among nurses of different genders” (FGD2-N)*.

*“I had a great experience where effective communication among team members, regardless of gender, led to a quick response to a critical situation” (FGD4-N).*

Nevertheless, NN-HCPs confirmed that both male and female nurses do present positive characteristics in clinical settings, including empathy, communication, teamwork in nursing care, and commitment to patient well-being, both excel in their roles and dedication to delivering high-quality care.

*“I’ve come to appreciate the importance of empathy, communication, and teamwork in nursing care, regardless of the nurse’s gender” (FGD1-NN-HCP).*

*“I think nurses regardless of gender identity, demonstrate remarkable teamwork and dedication to patient care” (FGD2-NN-HCP).*

*“What’s evident is the nurse’s shared commitment to patient well-being, which transcends any gender-related differences. Their collective focus on teamwork and patient-centered care is commendable” (FGD1-NN-HCP).*

*“In my experience, nurses’ dedication to delivering high-quality care remains consistent regardless of gender identity” (FGD2-NN-HCP).*

The clients also reported positive qualities of both male and female nurses in clinical practice, including attentiveness, good communication skills, sympathy, peacefulness, and response to clients’ needs

*“The nurses, both male and female, were generally more attentive and communicative than the doctor in that particular instance” (FGD3-C).*

*“It made me realize that nurses aside from them being either male or female show qualities like sympathy and communication well regardless of gender. I believe the majority of them possess that sense of peacefulness” (FGD3-C).*

*“I found that nurses, regardless of gender, were often more attuned to clients’ needs and concerns” (FGD3-C).*

### Subtheme 3.2: male nurses’ positive qualities and impact in clinical practice

Nurses themselves mentioned several positive qualities in male nurses, including being expert, calm, empathetic, attentive to details, skilled in analyzing data, skilled in recognizing changes in the patient’s condition, tend to be more direct and concise in their communication, solution-oriented communication style, ability to connect with young clients and their families, and adaptability in nursing.

*“He approached the situation with an orderly yet empathetic approach that immediately put all of us at ease and created a sense of trust and mutual respect. Our collaboration was perfect and I just loved his attention to detail regarding the patient’s condition. This nurse’s skill for analyzing data and recognizing any changes in the patient’s condition, was good which greatly contributed to the accuracy of our assessments and interventions” (FGD1-N).*

NN-HCPs on the other hand described male nurses as being more direct, taking a more collaborative approach, calm and confident, using the empathetic approach, more of what the rules state, too much of protocol, kind, more trusted, more compassionate, more task-oriented, and focusing on efficient execution of care plans.

*“Some male nurses being more direct and others taking a more collaborative approach” (FGD1-NN-HCP).*

*“Male nurses, on the other hand, can bring a sense of calm and confidence, especially in high-pressure situations” (FGD1-NN-HCP).*

*“Male nurses are more of what the rules state……too much of protocol” (FGD2-NN-HCP).*

*“I have seen male nurses who are more compassionate” (FGD3-NN-HCP).*

*“I think in working and collaborating with male nurses they show a more task-oriented approach, focusing on the efficient execution of care plans which sometimes eases our work” (FGD4-NN-HCP).*

In the same way, clients mentioned the positive qualities of male nurses in clinical settings, including delivering comprehensive health education, competent, having good communication skills, promoting patient comfort, being attentive, introducing themselves to clients to be known, and communicating the procedure before doing it

*“I am a hypertensive person with diabetes. As for me, a male nurse, in particular, stood out for the way he always attended to me when I came to the hospital. Truthfully speaking he answers all my concerns and explains my treatment plan clearly in a way that I feel so well understood and heard” (FGD4-C).*

*“I can recall a particular incident with a specific nurse who attended to me when I came to the hospital. This nurse was a man and I loved how he attended to me. Since I was undergoing a challenging procedure, the nurse not only displayed practical skills during a challenging procedure but also maintained a kind and comforting approach when dealing with me. I loved his ability to effectively communicate and it seriously had a big impact on my overall experience” (FGD3-C).*

*“Some male nurses were incredibly attentive and competent” (FGD3-C).*

*“The good experience was this male nurse who when I arrived received me and happily introduced himself I later learned that he was a student. But the way he handled me and explained every procedure I was supposed to do made me feel good. He even allowed me to ask questions if I had any. I have never been handled that way before. It left me feeling good” (FGD4-C).*

Nurses, NN-HCPs, and clients discussed the impact of the possessed positive qualities by nurses in the clinical areas. Having positive qualities result in positive effects. Nurses in different FGDs stated that when male nurses hold positive qualities in clinical facilities, it helps to put other nurses at ease, create a sense of trust, establish mutual respect, and promote the accuracy of assessments and interventions.

*“He approached the situation with an orderly yet empathetic approach that immediately put all of us at ease and created a sense of trust and mutual respect” (FGD1-N).*

*“This nurse’s skill for analyzing data and recognizing any changes in the patient’s condition, was good which greatly contributed to the accuracy of our assessments and interventions” (FGD1-N).*

NN-HCPs emphasized that when male nurses have positive characteristics in the clinical setting, create a spirit of collaboration within the team.

*“There have been instances where male nurses appeared more confident in voicing their opinions or suggestions during patient rounds which might sometimes affect the overall collaboration, especially when decisions need to be made collectively” (FGD2-NN-HCP).*

Clients also showed that the male’s positive qualities in clinical areas aid in preventing stress and promote good feelings among clients.

*“It seriously had a big impact on my overall experience. He removed my anxiety and I felt so peaceful” (FGD3-C).*

### Subtheme 3.3: male nurses’ negative qualities and effects on clinical practice

Nurses in different FGDs disclosed that male nurses are authoritative and with inappropriate comments on some issues that influence suboptimal outcomes in clinical settings.

*“Male nurses might be perceived as more authoritative” (FGD1-N).*

*“I encountered a situation where a male nurse made inappropriate comments about my uniform which I think had suited me well and putting in mind I have a big body which created an uncomfortable working environment” (FGD2-N).*

Nevertheless, NN-HCPs reveal that male nurses are assertive, love to be addressed as doctors by clients, and tend to spread rumors and gossip.

*“Some male nurses might be thought to be assertive, leading the clients to respond differently to medication adherence discussion” (FGD3-NN-HCP).*

*“Majority of male nurses would love to be addressed as doctors by clients and they do want to assume they are doctors and forget their roles as nurses who have specified roles and responsibilities” (FGD4-NN-HCP).*

*“I tend to see male nurses as people who one has to be careful with as they have the*.

*tendency to spread rumors” (FGD4-NN-HCP).*

Clients disclosed the negative qualities of male nurses in the clinical setting, especially their carelessness of patient’s pain during clinical procedures, lack of empathy, lack of caring habits, lack of professionalism, maintaining no privacy for clients, and dislike of being addressed as a nurse rather as a doctor

*“I was once admitted because I had a wound on my right leg, as I was being cleaned my wound by a male nurse who regardless of telling him I was in severe pain when he was removing the bandages, did not listen and pulled them painfully and I ended up enduring the pain. Then the dressing time (laughs nervously) arrived, he cleaned the wound very very roughly without giving me medication to help and after he had finished cleaning, he told me to pick up the used bandages and dispose of them myself” (FGD1-C).*

*“The nurse I interacted with, was a male, and I couldn’t help but wonder if his apparent lack of sympathy for my pain” (FGD1-C).*

*“I was admitted for a minor surgical procedure, and while most of the nursing staff were fantastic, there was this one male nurse who seemed disinterested and indifferent from the moment he walked in. He was brusque in his mannerisms, displaying a lack of empathy or concern for my well-being. During one instance, when I was experiencing severe pain and requested pain relief, he brushed it off, saying, ‘It’s not that bad, you’ll manage.’ It felt dismissive and insensitive, especially considering the discomfort I was in. What made matters worse was his lack of professionalism in maintaining my privacy. He didn’t properly close the curtain around my bed when attending to me, exposing me to unnecessary views from other clients and passersby in the hallway. It was embarrassing and made me feel vulnerable” (FGD3-C).*

*“Male seems more authoritative” (FGD3-C).*

*“I also had a bad experience still with a male professional who at first I thought was a doctor but as I was admitted one of the doctors called out “call for me that nurse. I looked and saw that he was the one being called, when he came letter to give me medicine I jokingly said that “…so nowadays you males are nurses which is good” I was kind of happy to see him as a male nurse since all the time I had been referring to him as a doctor. Weeeee……. I have never seen that anger, he thought I was making fun of him, threw my drug on my bed, and told me to medicate myself. To be sincere, I felt bad and from then till I was discharged I was treated very badly by some nurses in that ward and if I don’t remind them to give me drugs they don’t give me” (FGD4-C).*

Nurses, NN-HCPs, and clients reported that the negative qualities of nurses are linked to negative outcomes in the clinical setting. NN-HCPs indicated that when male nurses demonstrate negative qualities in clinical areas it affects clients, especially in medication adherence, and affects physicians in practice.

*“Majority of male nurses would love to be addressed as doctors, but sometimes it hampers my work. There is this scenario in which I was treating a patient and I told him to be doing physical exercise due to his weight and when I came back to make a follow-up, he told me Dr X had been very helpful and was assisting him whenever he wanted to walk around. Looking for Dr X, I found out he was a nurse that was when I noticed all the clients were referring to all male nurses in that unit as doctors……Later when I instructed the patient to take a certain drug, he literary said he would counter check with Dr X” (FGD4-NN-HCP).*

*“I also encounter the same problems in my unit and sometimes it hinders my work too. I think male nurses should accept that they are nurses and love their profession. Please note that not all male nurses have this character” (FGD4-NN-HCP).*

*“Some male nurses might be thought to be assertive, leading the clients to respond differently to medication adherence discussion” (FGD3-NN-HCP).*

Clients expressed the negative consequences that result from negative characteristics possessed or demonstrated by male nurses. The effects include clients’ frustration, and scared, it erodes patient’s trust in the healthcare system, makes clients hesitate to seek medical help, and leaves clients feeling uncomfortable.

*“It was incredibly frustrating. I spent my entire hospital stay so scared for every time my wound was to be cleaned and the 4 days I was there it was always being cleaned by the male nurses who were just the same. I hated the hospital and specifically any male nurse as I know it’s like they were being forced to work in the hospital” (FGD1-C).*

*“It eroded my trust in the healthcare system to some extent. I nowadays find myself hesitating to seek medical help unless necessary like today I have just come because I need the X-ray which I have been told is important to do it” (FGD1-C).*

*“I had an unfortunate encounter with a male nurse during my recent hospital stay that left me extremely uncomfortable” (FGD3-C).*

*“It felt dismissive and insensitive, especially considering the discomfort I was in” (FGD3-C).*

*“He didn’t properly close the curtain around my bed when attending to me, exposing me to unnecessary views from other clients and passersby in the hallway. It was embarrassing and made me feel vulnerable” (FGD3-C).*

*“I felt ignored, disrespected, and overall neglected under his care” (FGD3-C).*

### Subtheme 3.4: female nurses’ positive qualities and outcomes in clinical practice

Nurses in different FDGs acknowledged that female nurses possess positive qualities in hospitals. Several qualities were mentioned, including calmness, effective communication, team coordination, empathy, nurturing, detail in their communication, focus on emotional aspects during communication, building rapport, fostering open communication, and innovation in patient care.

*“Female healthcare providers often display more empathy and tend to focus on emotional aspects during communication” (FGD1-N).*

*“I’ve observed a different dynamic during patient consultations. Female healthcare providers, especially doctors and nurses, often excel in building rapport and fostering open communication” (FGD4-N).*

Yet, NN-HCPs revealed that female nurses are more empathetic, in a nurturing manner. They seem more understanding, often assign tasks, seek assistance, build rapport, establish trust with clients, and put emphasis on team collaboration. Moreover, they are compassionate, use a holistic approach to care, prefer open communication, and create comfortable and supportive environments for clients.

*“Female nurses I have interacted with show excellent teamwork skills, fostering collaboration and open communication within the healthcare team” (FGD4-NN-HCP).*

*“Some female nurses might approach the same scenarios with a more empathetic or nurturing manner” (FGD1-NN-HCP).*

*“Female nurses tend to excel in creating a comfortable and supportive environment for clients” (FGD2-NN-HCP).*

*“Some female nurses being seen as more understanding, influencing how clients engage in these conversations” (FGD3-NN-HCP).*

Clients in different FGDs had similar observations as nurses and NN-HCPs. They reported that female nurses deliver comprehensive education and navigation care to clients. They added that female nurses are sympathetic, empathetic, skilled, have good communication skills, involve clients in decision-making, compassionate

*“She not only explained to me my sickness at the time she also took time to thoroughly explain what I was supposed to eat and even offered to direct me to a seller who sells the food. Aside from that, she offered me an opportunity to explain what she had taught me to see if I had understood. I felt good” (FGD1-C).*

*“Female nurse would naturally be more sympathetic than a male nurse” (FGD2-C).*

*“One nurse, in particular, stood out. She was exceptionally skilled at balancing technical skills with genuine understanding while attending to me. This nurse showcased qualities that I would value in any healthcare professional, regardless of gender. Her communication was clear, she took the time to listen to my concerns, and she actively involved me in decisions about my care. It made me realize that these qualities are not exclusive to a particular gender but are essential components of effective nursing practice” (FGD1-C).*

*“One nurse, a female was good as she was a key part of my recovery after surgery. Her approach was remarkable. Not just in terms of ability to see my healing process but also in her ability to connect with me on a personal level” (FGD1-C).*

*“The nurse went beyond the regular care routine. She took the time to explain each step of my recovery process, making sure I felt informed and involved. Moreover, her empathetic and compassionate behavior was good” (FGD1-C).*

As it is with male nurses, nurses, NN-HCPs, and clients reported that the positive qualities possessed by female nurses corresponds to the positive outcomes at the clinical areas. Only one FGD for nurses appreciated the positive outcome from the positive qualities possessed by female nurses. They reported that female nurses enabled team dynamics during patient care.

*“I have a positive experience working with a female nurse during a particularly challenging night shift where we have multiple accident victims and short staffed. Her calm demeanor and effective communication made a significant impact on the overall team dynamics” (FGD1-N).*

On the other hand, NN-HCPs discussed that the positive qualities of female nurses remain the key factor influencing how clients engage in conversations.

*“Female nurses being seen as more understanding, influencing how clients engage in these conversations” (FGD3-NN-HCP).*

Similarly, Clients mentioned that the positive qualities of female nurses have resulted in clients feeling involved, supported, and comfortable.

*“Her empathetic and compassionate behavior made the hospital environment feel more supportive for me” (FGD1-C).*

*“I had a female nurse who made me feel comfortable during a sensitive exam. It made a big difference” (FGD2-C).*

### Subtheme 3.5: female nurses’ negative qualities and repercussions in clinical practice

Nurses in separate FGDs proclaimed that female nurses have negative characteristics in clinical areas that may have repercussions on patient care, such as being unable to correctly interpret clients’ instructions, having emotional reactions, and lacking confidence.

*“I think she had misinterpreted the clients’ instruction regarding a prescribed drug. When I told her she had misinterpreted the records she was so angry for no reason and told me that I was also a mere male nurse and should not bossy her” (FGD1-N).*

*“I’ve witnessed instances where we as female nurses are sometimes assumed to be less assertive or confident, especially in decision-making situations and it creates a challenging work environment, especially in cases where you make stern decisions regarding a staff behaviour” (FGD4-N).*

Furthermore, NN-HCPs mentioned only that female nurses are too argumentative in a clinical setting

*“Female nurses tend to be too argumentative” (FGD4-NN-HCP).*

Clients also uncovered some negative qualities of female nurses in the clinical areas. They mentioned that female nurses don’t communicate the procedure before implementation, possess poor communication skills, especially listening skills, are disinterested in clients’ needs, are impatient, do not respond to clients’ needs, delay in responding to patient demands, inefficient, less attentive, lack caring spirit, possess anger, doing tasks hastily like having other things to attend, and appeared visibly stressed

*“I had an unfortunate encounter with a female nurse that left me extremely uncomfortable. As someone who has always respected nurses, this experience shook my confidence. From the outset, the nurse appeared visibly stressed and overwhelmed. She didn’t announce herself when attending to me just ordered me to turn over and injected me not telling me what she had injected me with or whatever. I even didn’t want to ask her what it was (laughing) lest she rebuke me and seemed in a rush to attend to other tasks. Later when I tried to communicate my symptoms and concerns, she interrupted me multiple times, and it felt like she wasn’t even listening to me. I felt bad and here I was knowing that female nurses are more caring. This was my personal experience with this nurse though I have had better female nurses attending to me previously” (FGD2-C).*

*“There was one incident that bothered me. I was feeling that I needed more effort to breathe and I could feel the difficulty in breathing, and when I called for assistance, it took a considerable amount of time for her to respond. When she finally arrived, she seemed annoyed and didn’t take the time to understand my situation. Instead, she administered medication without explaining what it was for or how it would help” (FGD3-C).*

*“During my hospital stay, I noticed that this specific nurse was often impatient and seemed disinterested. Whenever I tried to ask questions about my treatment or express concerns about my symptoms, she brushed them off” (FGD3-C).*

*“Few female nurses seemed inefficient and less attentive” (FGD3-C).*

*“There was a female nurse who seemed like she did not care about my well-being, and the instructions regarding the drugs she had given me I did not well grasp and let me fill up the blanks. I tend to think maybe there were a lot of clients waiting to be attended but when I later saw that she was free, I went back to her for her to explain further but she angrily brushed me off. Luckily I went to a pharmacy shop and the shopkeeper explained to me” (FGD4-C).*

Clients disclosed the interdependence of female nurses’ negative qualities and negative impact at the clinical settings. Only clients in FGDs reported the negative outcomes that are likely to occur when female nurses possess negative qualities in clinical settings. Among the effects is clients feeling neglected and a burden. It also erodes clients’ trust in the healthcare system.

*“It made me feel like I was being a burden rather than someone in need of care” (FGD3-C).*

*“It was disheartening. I came to the hospital seeking help and understanding, but I felt neglected and dismissed. It eroded my trust in the healthcare system and left me questioning the quality of care provided in such settings” (FGD3-C).*

### Theme 4: positive value of male nurses in clinical facilities from colleagues and clients

Male nurses seem to be much more valued compared to female nurses. They are valued by their colleague nurses and by clients cared for. The fourth theme had two sub-themes (Table 2), and the detailed information are provided below.

### Subtheme 4.1: value of male nurses by colleague

It was reported that the suggestions given by male nurses are more likely to be acknowledged and accepted than what is suggested by female nurses. Moreover, the recovery of clients is associated with male nurse’s care despite the involvement of female nurses during the care.

*“I’ve had instances where my suggestions are overlooked during team discussions, and if the suggestion comes from a male colleague who is a nurse it is accepted” (FGD3-N).*

*“There have been instances where male colleagues were given more credit for successful patient outcomes, even when it was a collaborative effort. There was a time when we worked tirelessly on a patient who had an ulcerative wound cleaning and making sure the healing process was as per the standards. After 2 months, the patient started walking and the wound was healed. During the morning meeting., one male doctor openly praised a male staff in the same ward I was in for making sure the patient did not turn up with an infection” (FGD3-N).*

*“I’ve had instances where my suggestions were sometimes overlooked during team discussions, and it seemed that my male colleagues received more immediate acknowledgment” (FGD4-N).*

### Subtheme 4.2: value of male nurses from clients

Male nurses are more trusted in the healthcare setting as compared to females and receive exceptional respect from their counterparts.

*“I’ve had instances where my fellow male nurse colleagues received more immediate trust and respect from clients, even if we had similar qualifications. This behavior from the clients makes me so dissatisfied and see a need to educate them that whether male or female, a nurse is a nurse” (FGD2-N).*

### Theme 5: Different cooperation between male and female nurses in clinical settings

Nurses and clients in the FGDs discussed the interaction of male and female nurses in the clinical areas. Some mentioned the presence of good interaction and others reported poor interaction. They emphasized that the status of interaction among these nurses has a direct impact on nursing care. The fifth theme had two sub-themes (Table 2), which are elaborated on below.

***Subtheme 5.1: Good interaction between male and female nurses***.

Clients discussed their experiences and observations, mentioning that male and female nurses have good teamwork during nursing care and are in unity.

*“I have noticed a sense of unity among nursing and it doesn’t seem to be influenced by gender. Especially in their social events and celebrations” (FGD2-C).*

*“I’ve witnessed instances where nurses, regardless of gender, have shown great teamwork” (FGD3-C).*

### Subtheme 5.2: poor interaction of male and female nurses in clinical practice

There is poor interaction between male and female nurses especially when female nurses don’t want male nurses to provide any advice, which automatically distorts the whole coordination of care.

*“I had when I was working with a female nurse in the ICU, thank God she is not among us here. I think she had misinterpreted clients the clients’ instructions regarding a prescribed drug. When I told her she had misinterpreted the records she was so angry for no reason and told me that I was also a mere male nurse and should not boss her Later our communication was strained, and it affected the overall coordination of care and luckily, I was transferred to the Medical Unit” (FGD1-N).*

### Theme 6: mixed perspectives towards clinical competencies across nursing gender

Through different FGDs by nurses, two subthemes emerged in the study including a negative perspective of male nurses’ clinical competency and a negative perspective of female nurses’ clinical competency. The sixth theme had two sub-themes (Table 2), which are elaborated on below.

### Subtheme 6.1: negative perspective towards male nurses’ clinical competency

Female nurses have been holding negative perspectives of the competencies of male nurses as they doubt whether male nurses can deliver the needed care to the nursing mothers or even demonstrate empathy when giving care to clients.

*“I know a lot of incidences where my ward in charge assumes that I can’t offer counseling, especially to nursing mothers simply because of my gender. I also remember during a team meeting, openly asked if I know how to display empathy, I felt bad although it sounded like a joke but still it had an underlying meaning” (FGD1-N).*

### Subtheme 6.2: negative perspective towards female nurses’ clinical competency

Even though female nurses are skilled, they are perceived to be unable to handle emergency issues, especially for clients in need of critical care.

*“I can testify to this as I have personally witnessed an emergency at EMD and the female nurse was the second in charge as the in-charge male was not around, she was assumed to be less capable of handling emergencies simply because of her gender and a male nurse in charge of a particular unit was tasked to handle the situation. Despite the female nurse being experienced and skilled, there was an automatic assumption that a male nurse would handle critical cases better” (FGD1-N).*

### Theme 7: perspective towards gender diversity in nursing

Several participant nurses, NN-HCPs, and clients in different FGDs had different perspectives in regard to gender diversity in nursing. Their perspectives are categorized into; the need for diversity in nursing, the need to address gender biases in nursing, the relationship between nursing gender and competence, and the distribution of opportunities. The seventh theme had four sub-themes (Table 2), which are detailed elaborated on below.

### Subtheme 7.1: the need for diversity in nursing

Nurses have indicated that diversity in nursing is good, and there is a need to embrace it. It is associated with the strength within the team and creates a suitable environment in the delivery of healthcare.

*“It’s about embracing diversity as a strength within the team” (FGD1-N).*

NN-HCPs confirmed that there is a demand for gender diversity in nursing. It should be appreciated, respected, embraced, celebrated, and valued due to its benefits.

*“Nursing practices should prioritize a collaborative environment where diverse approaches are valued, focusing on delivering the best patient-centred care” (FGD4-NN-HCP).*

*“I have also come to appreciate the diverse skill sets and approaches that both male and female nurses bring to patient care” (FGD3-NN-HCP).*

*“The gender diversity within nursing should be celebrated” (FGD1-NN-HCP).*

*“It is important to embrace and respect diverse styles and strengths from different healthcare providers regardless of gender” (FGD2-NN-HCP).*

*“My limited interactions with nurses of different genders have shown me the significance of acknowledging and valuing diverse communication styles regardless of gender” (FGD2-NN-HCP).*

Meanwhile, clients supported the views of nurses and NN-HCPs by showing that having gender diversity in nursing is a good asset and brings different tastes to a clinical setting.

*“I also think the hospital needs to have both male and female nurses from different parts of Tanzania to bring in different tastes” (FGD4-C).*

*“Both male and female in the nursing profession is an asset” (FGD4-C).*

### Subtheme 7.2: the demand to address gender biases in nursing

Due to the significance of gender diversity in nursing, the participants indicated that the effort to address challenges as gender diversity in nursing is inevitable. When gender bias is overcomed, patient care will be optimal.

*“There is the need for continuous efforts to challenge and overcome gender biases in healthcare” (FGD1-N).*

*“It emphasizes the importance of recognizing and addressing biases to ensure that every nurse, irrespective of gender, has the opportunity to contribute fully to patient care” (FGD1-N).*

*“Address any concerns related to gender or other factors that might impact patient care” (FGD2-N).*

### Subtheme 7.3: the relation between nursing gender and competence

Te majority of nurses in different FGDs emphasized that nurse performance is not determined by nurse gender but rather by competence, professionalism, empathy, effective communication, expertise, dedication to work, and commitment.

*“I believe competence and professionalism are crucial in healthcare, regardless of gender” (FGD1-N).*

*“There is a need for a shift towards recognizing and valuing competence and skills over traditional gender roles” (FGD1-N).*

*“Nursing excellence is not bound by gender (FGD1-N).*

*“It is important to look beyond gender stereotypes in nursing. Positive collaborations*.

*showcased that competence, empathy, and effective communication are essential qualities, irrespective of gender” (FGD2-N).*

*“Qualities like empathy and adaptability are crucial in healthcare, and they are not bound by gender” (FGD2-N).*

In the same line, NN-HCPs highlighted that expectations from nurses should be based on individual capabilities and skills rather than gender stereotypes. Moreover, NN-HCPs were of the opinion that qualities like compassion, expertise, effective collaboration, and understanding aren’t tied to any specific gender. The high level of professionalism and dedication from nursing personnel focuses more on competency rather than gender-specific traits. They emphasized that gender doesn’t determine competency but rather contributes to a rich diversity in nursing practice. Therefore, understanding the importance of individual strengths and skills rather than gender in nursing is essential.

*“I’ve also learned that expectations should be based on individual capabilities and skills rather than gender stereotypes” (FGD1-NN-HCP).*

*“I think we need to appreciate the diverse strengths of nurses, irrespective of gender and acknowledge individual skills and communication styles rather than categorizing based on gender as I expect a high level of professionalism and dedication from nursing personnel focusing more on competency than gender-specific traits” (FGD2-NN-HCP).*

*“My experiences have shown me that nursing is a field where competence and compassion matter more than gender. While I acknowledge different communication styles, I’ve come to expect professionalism and dedication from nurses, irrespective of gender” (FGD3-NN-HCP).*

The majority of clients in different FGDs show that gender in nursing does not predict excellence in the delivery of care. They insisted that good communication skills, caring, delivery of nursing services, quality of care, and compassion are not linked to gender, but rather individual competency.

*“I’ve had positive experiences with both male and female nurses in the past, but it made me more aware of the need for providing good nursing service irrespective of gender” (FGD2-C).*

*“I believe they have the qualities to take care of all clients, regardless of whether the nurse is male or female. It’s about the personal connection and the ability to understand the unique needs of the patient” (FGD2-C).*

*“I came to see nursing practice as a profession where the emphasis is more on the individual’s skills, rather than their gender” (FGD3-C).*

*“It’s about the individual’s ability to connect with me on a personal level that will make me feel comfortable that I am being properly attended to” (FGD4-C).*

*“Positive interactions with nurses of both genders have shown me that competence and*.

*communication are important irrespective of gender. We have heard how a male nurse was good and how another male nurse was bad and vice versa with the female. I think practice should not be tied to gender” (FGD4-C).*

### Subtheme 7.4: distinct importance of nursing gender diversity to patients and nurses

The availability of both male and female nurses at the healthcare facility has the benefits to clients, especially, creates a supportive environment, allowing clients to choose the preferred gender during the care. It influences the clients to express their needs freely, which promotes the patient’s comfortable experience.

*“Most of the time and within our unit we have noticed that clients sometimes feel more comfortable discussing certain concerns with healthcare providers of the same gender. This awareness in our team allows us to ensure clients’ preferences are respected” (FGD1-N).*

*“Creating a diverse and inclusive healthcare environment is important. Having a team with a variety of genders allows clients to have choices and may contribute to a more comfortable experience” (FGD1-N).*

*“Regardless of gender identity, a collaborative approach ensures that diverse skills and perspectives are utilized for the benefit of the patient” (FGD1-N).*

*“I’ve also encountered situations where clients, especially in diverse cultural contexts, had strong preferences for healthcare providers of the same gender. Being aware of and accommodating these preferences is crucial for providing patient-centered care” (FGD2-N).*

*“I’ve seen instances where having a diverse team, including both male and female healthcare providers. Different perspectives contribute to more comprehensive and well-rounded patient care” (FGD4-N).*

*“I want to highlight a positive experience where our team, consisting of both male and female nurses, collaborated exceptionally well during a challenging situation, and ended up saving the patient’s life. Indeed, without working together, we would have lost the patient” (FGD4-N).*

*“Positive experiences have shown me the strength of diversity in improving patient outcomes” (FGD1-N).*

Consistently, the NN-HCPs indicated that varied perspectives and diverse communication styles contribute significantly to patient care, contribute to a well-rounded and patient-focused care environment, address the diverse needs of clients, impact patient care, and promote a holistic approach to patient care.

*“Witnessing the diverse strengths and approaches of both male and female nurses has reinforced my belief in the importance of a gender-diverse nursing workforce. It’s evident that these varied perspectives and communication styles contribute significantly to patient care” (FGD1-NN-HCP).*

*“I think recognizing the unique strengths each gender brings to nursing practice has reinforced the importance of an inclusive and diverse nursing team as it’s with these varied approaches that contribute to a well-rounded and patient-focused care environment” (FGD1-NN-HCP).*

*“Often results in comprehensive care plans that address the diverse needs of clients” (FGD2-NN-HCP).*

*“Both male and female nurses bring unique perspectives and strengths that positively impact patient care regardless of their communication styles” (FGD3-NN-HCP).*

*“Each nurse, regardless of gender, brings valuable perspectives that contribute to a holistic approach in patient care” (FGD3-NN-HCP).*

*“Their ability to work together, leveraging individual strengths, fosters an environment conducive to excellent patient outcomes” (FGD2-NN-HCP).*

Several nurses’ discussions in different FGDs indicate that gender diversity in nursing has many advantages for nurses themselves. It enhances the overall team dynamic, creates a supportive work environment, learn from each other, shares insights, promotes collaboration, enhances inclusive decision-making, influences personal comfort, and ease of communication and coordination. Moreover, gender diversity fosters problem-solving approaches and brings different perspectives.

*“Having both male and female colleagues has created a supportive work environment. We learn from each other, share insights, and that friendship enhances the overall team dynamic” (FGD1-N).*

*“Positive experiences have shown the benefits of inclusive decision-making, showcasing that everyone’s input matters” (FGD1-N).*

*“In my experience, diverse teams with a mix of male and female nurses bring different perspectives and problem-solving approaches” (FGD2-N)*.

*“Having diverse healthcare teams can contribute to a more understanding and inclusive environment” (FGD3-N)*.

*“I’ve been part of teams where mutual respect and support, regardless of gender, created an enjoyable work environment” (FGD4-N)*.

Similarly, the NN-HCPs, reported that gender diversity in nursing enhances the overall team dynamic, better teamwork, enriching the overall healthcare delivery, ability to connect with clients, quality care, brings unique perspectives and strengths, enriches nursing practice, brings valuable perspectives, diverse skill sets and communication styles to their practice.

*“I think the gender mix among nurses can enhance the overall team dynamic” (FGD1-NN-HCP)*.

*“Gender diversity in nursing can foster better teamwork and understanding” (FGD1-NN-HCP)*.

*“Male and female nurses offer, enriching our overall healthcare delivery. It’s also good to note that gender can influence team dynamics among nurses” (FGD1-NN-HCP)*.

*“I think from my experience, gender dynamics with nurses can indeed play a role in collaboration” (FGD2-NN-HCP)*.

*“From my experience, I think that gender diversity among nurses enhances the collaborative nature of healthcare teams. Both male and female nurses bring unique perspectives and strengths” (FGD3-NN-HCP)*.

*“I think nurses of different genders bring diverse skill sets and communication styles to their practice” (FGD4-NN-HCP)*.

### Theme 8: preferences of nurse’s gender, reasons, and opinion towards gender preferences

Nurses, NN-HCPs, and clients in different FGDs were asked about issues related to nursing gender preferences. NN-HCPs revealed their experiences on which clients had a preference of a specific nurse gender to be cared by. On the other hand, nurses in FGDs reported that when they sought medical attention, they had mixed reactions on which gender they prefer to care for them. Meanwhile, clients expressed their preferred gender nurse when seeking medical attention. Therefore, seven subthemes emerged in this area, including; having no gender preference for nurses, having a preference for nurses of a specific gender, having a preference for nurses of the same gender, having a preference for nurses of the opposite gender, reasons for gender preferences, the challenge of meeting clients’ preferences through nurse’s gender diversity, and different opinion about gender preferences. The eighth theme had seven sub-themes (Table 2), which are elaborated on below.

### Subtheme 8.1: having no gender preference for nurses

One participant in a single FGD mentioned that clients had no preference for nurse gender rather they felt comfortable with any gender.

*“I have observed situations where clients didn’t have a preference and were comfortable with healthcare providers of any gender” (FGD4-NN-HCP).*

Some clients in FGDs indicated that they have never deliberately chosen to have a specific gender when seeking medical help.

*“I have never had a conscious preference for a nurse of a specific gender. To me, it’s always been about the individual’s competence, communication skills, and ability to connect with clients. I believe these qualities are not confined to any particular gender” (FGD1-C).*

*“No, gender has never been a deciding factor for me. I look for qualities such as kindness, attentiveness, and professionalism. These attributes can be found in nurses of any gender, and that’s what influences my comfort and trust in their care” (FGD2-C).*

*“I haven’t had a specific preference for a nurse’s gender. For me, it’s always been about who is around to help me” (FGD3-C).*

Consistently, the majority of nurses in the FGDs show no preference for gender nurses but rather accept any available nurse with the required competencies.

*“I also have never had any preference for a nurse’s gender. I believe competence*.

*and professionalism is crucial in healthcare, regardless of gender” (FGD1-N).*

*“Now that I am a nurse it will seem funny if I have a preference because I know all or majority of us have gone through the same educational training. But as a patient, I think I never had a specific preference for a nurse’s gender. What matters most to me is their competence, communication skills, and ability to provide compassionate care” (FGD2-N).*

*“I’ve never had a specific preference for a nurse’s gender as what I look for is competence, communication skills, and ability to provide compassionate care” (FGD3-N).*

### Subtheme 8.2: having a preference for nurses of a specific gender

Clients feel more at ease discussing sensitive topics with a nurse of the same gender and when they are receiving instructions or explanations about their medications.

*“I’ve encountered clients who had preferences for a nurse of a specific gender while receiving instructions or explanations about their medications” (FGD1-NN-HCP).*

*“I have come to understand that clients might sometimes express preferences for nurses of a specific gender” (FGD2-NN-HCP).*

*“Clients feel more at ease or open to discussing certain health issues with nurses of their preferred gender, which can impact the overall care experience” (FGD3-NN-HCP).*

One client mentioned being comfortable or understood when a nurse of a certain gender is available and providing care.

*“I found myself paying more attention to the gender of the healthcare professionals involved in my care. It made me consider whether I felt more comfortable or understood when the nurse was of a certain gender. It’s not that I believe one gender is inherently better, but this experience has made me more attuned to the dynamics and the impact it might have on my experience” (FGD1-C).*

### Subtheme 8.3: having a preference for nurses of the same gender

NN-HCPs in different FGDs expressed their experiences where they have observed clients preferring to receive care from nurses of the same gender. Their preferences are based on when they have sensitive issues to share with healthcare provider

*“Some female clients might feel more comfortable discussing certain personal or sensitive issues with female doctors or nurses” (FGD2-NN-HCP).*

*“The clients may feel more at ease and communicate more openly with a nurse of the same gender” (FGD3-NN-HCP).*

*“There were instances where clients seemed more persuaded to share personal details with nurses of the same gender, which could potentially impact the information conveyed to the medical team” (FGD3-NN-HCP).*

*“I have encountered clients who felt more at ease discussing sensitive topics like personal hygiene or reproductive health with a nurse of the same gender” (FGD4-NN-HCP).*

*“There might be a preference for same-gender care providers during intimate procedures or examinations due to modesty or religious reasons” (FGD4-NN-HCP).*

Clients in different FGDs reported that they prefer nurses of the same gender when discussing personal issues. They feel comfortable and receive sympathy.

*“As a female, during discussions about the more personal aspects of my well-being, I somehow felt more comfortable talking to female nurses. It was my understanding that they might be more open to these discussions” (FGD1-C).*

*“As a male, I’ve sometimes felt more comfortable with male nurses during certain personal care situations. It’s not about competence, but more about feeling understood. Nevertheless, I’ve had exceptional care from nurses of both genders” (FGD3-C).*

Nurses in FGDs revealed their preference and desire to have nurses of the same gender when seeking medical attention. They too mentioned their observation where clients deliberately ask the nurse of the same gender.

*“I must admit that in certain situations, I’ve felt more at ease with healthcare providers of the same gender. It’s not a strict preference, but there are instances where discussing personal health matters feels more comfortable with someone of the same gender” (FGD3-N).*

*“I’ve had female clients who explicitly requested a female nurse for intimate procedures she was undergoing and some discussions about personal health matters” (FGD3-N).*

*“I recall a scenario where a female patient specifically requested a female nurse for a sensitive procedure. The patient expressed discomfort with a male nurse due to personal reasons. While it wasn’t a reflection on competency, the patient’s comfort was paramount” (FGD4-N).*

### Subtheme 8.4: having a preference for nurses of the opposite gender

Clients unzipped the fact that they prefer nurses of the same gender, making them feel at ease and comfortable for certain personal discussions.

*“I would unconsciously seek out a female nurse when going to the hospital before opting for the available person to attend to me” (FGD1-C).*

*“I do notice that I tend to feel more at ease with female nurses. Maybe it’s because I’m used to it” (FGD1-C).*

*“If given an opportunity I choose female nurses” (FGD4-C).*

It was identified that some nurses wish to be cared for by nurses of the opposite gender.

*“On a personal level, if seeking medical help for me, even if I am a male nurse, I feel more comfortable discussing personal health issues with a female nurse. It just comes naturally for me” (FGD4-N).*

### Subtheme 8.5: reasons for gender preferences

Some clients said that their preferences for nursing gender are when they have personal discussions, sensitive issues to discuss, and during sensitive clinical procedures.

*“Well, there was a time when I was going through a particularly sensitive health issue, and I found myself leaning towards having a female nurse. I think it was because I assumed she might understand the emotional aspect better” (FGD4-C).*

Nurses in different FGDs discussed that nurses or clients who choose a certain gender whether similar or opposite, are in certain situations where discussing sensitive issues is involved, they seek comfort, looking for someone with whom it is easy to share their concerns and ask questions, and due to cultural issues.

*“I can relate to having a preference, especially in certain situations where discussing sensitive issues is involved. I found that having a female nurse created a more comfortable environment for me to openly communicate about personal health matters” (FGD1-N).*

*“As for me, regardless of being a male nurse, I have in some situations, felt more comfortable discussing personal health issues with a female nurse. It was more about personal comfort and ease of communication” (FGD1-N).*

*“I’ve had female clients who explicitly requested a female nurse for intimate procedures she was undergoing some discussions about personal health matters” (FGD3-N).*

*“Clients seem to feel more at ease sharing their concerns and asking questions with female providers” (FGD4-N).*

The majority of clients discussed their nursing gender preference based on competency. They revealed that they prefer a nurse with the required competencies, caring elements, communication skills, confidence in delivering care, understanding clients’ needs, kind, attentive, professional, open in discussion, and available with empathy rather than gender.

*“I don’t think I’ve ever had a preference based on gender. For me, it’s more about the nurses on duty and specifically how they communicate and care” (FGD1-C).*

*“I don’t think I’ve ever had a preference based on gender. For me, it’s always been about the nurse’s competence and how comfortable they make me feel” (FGD4-C).*

Nevertheless, nurses in FGDs reported that those with no gender preference focus on professionalism, expertise, competence, communication skills, and ability to provide compassionate care.

*“The most important factors for me are their professionalism and expertise in delivering quality healthcare” (FGD2-N).*

*“I look for competence, communication skills, and ability to provide compassionate care” (FGD3-N).*

Most of the clients in different FGDs insisted that their preferences are tied to the service/care needed. For instance, preference may be tied to the demand for emotional support, a sense of security, caring service, the demand of peacefulness, help, empathy, and recovery services.

*“I did and do have a preference. It was during a particularly sensitive situation, and I felt more comfortable with a female nurse. I think it was about emotional support” (FGD1-C).*

*“It was a personal health issue, and I felt like a female nurse would understand the emotional aspect better. It wasn’t that I thought a male nurse couldn’t provide excellent care, but it was about connecting on a different level” (FGD1-C).*

*“I guess I might have unconsciously preferred a male nurse in certain situations. Maybe because I associate their presence with a sense of security” (FGD1-C).*

*“I tend to seek out female nurses if I am in the hospital because I believe they are sympathetic” (FGD4-C).*

*“It’s about feeling understood on a deeper level. I assumed a female nurse might sympathize more with the emotional aspects of my situation” (FGD4-C).*

*“I had surgery, and I felt more at ease having a female nurse during the recovery process. It just felt less awkward for me” (FGD4-C).*

Clients mentioned culture as the factor motivating them to choose a nursing gender when seeking medical interventions. Some added that they grew up in an environment with gender sensitivity which has influenced their routine preference of nursing gender.

*“I have grown up in a home where discussing personal matters with someone of the opposite gender can be uncomfortable, I’ve often preferred a nurse of the same gender” (FGD1-C).*

*“I have found myself leaning towards female nurses because I grew up discussing*.

*health matters primarily with women in my family. There’s a familiarity and comfort*.

*in discussing personal issues with someone of the same gender” (FGD1-C).*

*“I am from a conservative background, so I will always feel more comfortable with female nurses for certain personal discussions. It’s something about the cultural thing for me” (FGD3-C).*

NN-HCPs in different FGDs disclosed what influenced clients to have a preference for nursing gender (whether same or opposite gender), including cultural considerations and religious reasons.

*“I think the preference often arises due to cultural or personal reasons, where individuals might feel more comfortable discussing certain health issues with someone, they perceive may have an understanding from a shared gender perspective” (FGD4-NN-HCP).*

*“I think it might be related to cultural considerations. For instance, in certain cultures, clients might feel more at ease discussing sensitive matters with healthcare providers of the same gender” (FGD4-NN-HCP).*

Some NN-HCPs unzipped the fact that they prefer a certain gender in nursing because of the expectation of comfort.

*“It seems these preferences are often linked to personal comfort levels and the nature of the information being shared” (FGD1-NN-HCP).*

*“I have come to understand that clients might sometimes express preferences for nurses of a specific gender based on personal comfort” (FGD2-NN-HCP).*

*“Maybe they may trust the person so they feel comfortable and with to share with them whatever that is bothering them” (FGD4-NN-HCP).*

NN-HCPs reported that clients who don’t choose a specific nursing gender are due to the fact they trust the healthcare system, making them not worry about any gender.

*“Some clients trust in the healthcare system and know that service is service regardless of the provider’s gender” (FGD4-NN-HCP).*

NN-HCPs said that the individual past experiences in seeking medical intervention might influence the person to have a preference for nurses a of certain gender.

*“I tend to think that it might depend on the individual’s patient’s personality and maybe some past experiences where he or she was either blamed for choosing a provider” (FGD4-NN-HCP).*

### Subtheme 8.6 the challenge of meeting clients’ preferences through nurse’s gender diversity

Nurses in FGD revealed that even though patient preference is significant, it is hindered by a shortage of nursing workforce.

*“Clients’ preferences are respected although not always as sometimes with a hectic day and limited staff available, we sometimes fail to attain our goal” (FGD1-N).*

Meanwhile, NN-HCPs supported that even though it has been reported that preferences of nursing gender by clients exist, it is challenged by a shortage of nursing workforce.

*“Clients might request nurses of a specific gender for personal care, and if there’s a shortage of nurses of that gender, it could impact how responsibilities are distributed” (FGD4-NN-HCP).*

*“I think addressing patient preferences related to gender requires a bigger approach putting in mind the scarcity of the workforce” (FGD1-NN-HCP).*

### Subtheme 8.7 different opinions about gender preferences

Different NN-HCPs in different FGDs gave their suggestions and opinions about clients’ preferences for nursing gender. They suggested that nurses should consider and respect clients’ preferences, and should create an environment for clients to express their demands for nursing gender.

*“It’s crucial to respect these preferences when feasible without compromising the quality of care. I think we can facilitate this by having open discussions with clients, understanding their preferences, and making reasonable accommodations whenever possible” (FGD1-NN-HCP).*

*“We as healthcare providers should prioritize a patient-centered approach by actively listening to patient’s preferences regarding the gender of their healthcare providers. We also need to create an environment where clients feel comfortable expressing their preferences without judgment” (FGD1-NN-HCP).*

*“Respecting these preferences when feasible contributes to patient-centered care” (FGD2-NN-HCP).*

Most nurses in different FGDs indicated the value of clients’ or nurses’ preferences of nursing gender. Most of them insisted that preference is a good thing, and several strategies should be reinforced to promote preference culture. Healthcare providers need to encourage clients to state their preferences and there should be an environment where clients may feel comfortable expressing their preferences.

*“I think open communication is key. Providers should encourage clients to express their preferences and concerns regarding gender” (FGD-1).*

*“I still believe that healthcare providers can initiate conversations with clients about*.

*their preferences, ensuring a comfortable space for them to express any concerns or preferences related to the gender of their healthcare team” (FGD-2).*

Most of the clients in different FGDs provided different opinions, suggestions, and comments about gender preferences. They mostly focused on strategies to encourage patient’s preferences for nurses. They emphasized that healthcare providers need to ask about clients’ preferences during admission, and healthcare providers and clients should be trained to understand the benefit of clients’ preferences in clinical areas, there is a need to create an environment for open communication and make clients comfortable to express their preferences. Moreover, they insisted that patient’s preferences need to be respected

*“Maybe healthcare providers could ask about our preferences during intake or admit processes, allowing us to express our comfort zones” (FGD1-C).*

*“I think the key is open communication. Healthcare providers can create an environment where clients feel comfortable expressing their preferences” (FGD1-C)*.

*“The hospital should also create an environment where we feel comfortable expressing our preferences without judgment is important” (FGD2-C).*

*“Training could be beneficial for healthcare professionals so that they can provide care effectively” (FGD1-C).*

*“I believe the healthcare providers can communicate and provide education clearly emphasizing that all staff, regardless of gender, are trained to provide high-quality care. This will help build trust with us” (FGD2-C).*

*“Education both us as clients and the healthcare providers to understanding that good care is not determined by gender, providers” (FGD2-C).*

*“If clients are informed that they have the right to express gender preferences and that the healthcare team will do their best to accommodate, it could lead to more open discussions” (FGD1-C).*

*“Perhaps asking clients about their preferences in a respectful way could be an option without making assumptions” (FGD2-C).*

*“The healthcare providers should consider giving us the choice to specify our gender preferences for care, if possible without being pointed out as being bad” (FGD2-C).*

## Discussion

The data were collected from 59 participants found at the four different hospitals in Dar es Salaam. A total of 12 FGDs. were conducted, comprised of nurses, non-nurse healthcare providers (physicians, laboratory technicians, scientists, and pharmacists)., and clients. The discussion is based on the identified themes.

### ① variations of male and female nurses in communication

It was found that male and female nurses differ in how they communicate, especially when communicating with clients and colleagues. Their difference is in the communication style adopted and is highly observed when discussing medications. For instance, when communicating with clients, female nurses tend to provide detailed information and demonstrate empathy and nurturing elements. While, male nurses are brief, concise, more direct, and assertive. It was reported whatever differences when discussing with clients, both communication styles are helpful and effective. The finding is supported by the study, which stated that male nurses are forthright and to the point, while female nurses consider the world as a network of connections and solidarity, leading their communication to be exhaustive and supportive [36]. Male nurses apply humor and establish mutual trust, but female nurses use touch during communication to demonstrate empathy and calm the patient [37]. Meanwhile, when female nurses are receiving instructions from colleagues they ask for more details, but male nurses don’t. While participants indicate that gender in nursing is the source of communication differences, others indicated that communication is not linked to nursing gender, rather personality, experience, and communication skills, majority. The previous study indicate that the communication difference is neither embedded to gender nor biological, but rather behavior, race, ethnicity, and socioeconomic background [38].

### ② differences between male and female nurses in carrying out leadership roles

It was found that male nurses use an authoritative leadership style while female nurses assume a democratic leadership style. Both leadership styles are good and recommended in the institutions. The previous study stated that an authoritarian style of leadership directs staff towards specific tasks they must obey and it promotes a highly structured work environment [39]. Male nurses seem to use an authoritative style because most of them are working in emergency demanding situations that require fast responses to clients, otherwise the delay may lead to a patient’s death. Therefore, in emergency cases decisions are made by fewer people and that makes authoritative an option. It is aligned with the literature, recommending that authoritarianism fits better in emergencies where quick action is required everyone works towards the same goal, and decisions do not need to be discussed at length [40]. In contrast, female nurses seem to use a collaborative style because most of them work in non-emergency units. Their cases do not demand quick decisions, that’s why they end up using a democratic leadership style. Adopting a certain style depends on personality, belief system, company culture, employee diversity, personality traits, level of control, organizational structure, and experience [41].

### ③ divergent clinical qualities and outcomes across nursing gender

Both male and female nurses reported having common positive qualities like good collaboration, communication skills, empathy, work commitment, attentiveness, sympathy, peacefulness, responding to clients’ needs, calmness, compassion, and promoting patient comfort. These shared qualities might have been obtained from their educations, which are well stipulated within nursing curricula. It is supported by previous literature denotes that nursing curricula contain different information to improve communication skills, teamwork, skills, attitude, and knowledge [42].

Nevertheless, male nurses have unique positive qualities that female nurses don’t have, including being expert, calm, attention to detail, skill for analyzing data, skill for recognizing any changes in the patient’s condition, tending to be more direct and concise in their communication, solution-oriented communication style, ability to connect clients and their families, confident, more of what the rules state, too much of protocol, kind, more trusted, more task-oriented, focusing on the efficient execution of care plans, delivering comprehensive health education, competent, attentive, introduce themselves to the patient to be known and communicate the procedure before doing it. The unique caring behavior of male nurses has been reported by previous literature that they are respectful, considerate, good listener, unbiased, and supportive [43]. Additionally, male nurses are polite and courteous during service delivery [44]. They also form trusting relationships in the clinical setting compared to their counterpart female nurses [45].

In contrast, female nurses reported to have their unique positive characteristics that are not found in male nursing, including nurturing, detail in their communication, focus on emotional aspects during communication, building rapport, fostering open communication, innovation in patient care, more understanding, often assign tasks, seek assistance, establish trust with clients, use a holistic approach to care, prefer open communication, create supportive environments for clients, deliver comprehensive education and navigation care to clients, and involve clients in decision making.

All positive qualities for female nurses and male nurses are good.Most of their qualities should have been learned from their professional education and clinical experiences, but some may have been rooted in their natural gender. For instance, some male nurse’s qualities are dominated by manhood like confidence, too many rules, and comprehensiveness, while female nurses are detailed in communication and deliver holistic care. Since all positive qualities in both genders are important, every nurse regardless of gender should possess them.

It has been found that positive qualities for both nursing genders have a positive outcome while their negative qualities influence negative effects in the clinical areas. For instance, the positive qualities of male nurses put other nurses at ease, create a sense of trust, establish mutual respect, promote the accuracy of assessments, promote effective interventions, create a spirit of collaboration within the team, prevent stress, and promote good feelings among clients. Moreover, the positive qualities of females enable team dynamics during patient care, a key factor influencing how clients engage in the conversations, and make clients feel involved, supported, and comfortable. Therefore, the positive qualities of whatever gender has an outcome on colleagues and clients, make clients able to receive optimal care and enhance team collaboration. The finding is supported by the previous study by Danwil [43, 46].

Regarding negative qualities, no reported shared negative qualities for male and female nurses. It is found that every gender has unique negative characteristics. For instance, male nurses are reported to be authoritative, and assertive, love to be addressed as doctors by clients and dislike to be addressed as nurses rather as doctors, tend to spread rumors, and gossip, careless of patient’s pain during clinical procedures, lack of caring habit, and maintaining no privacy for clients. On the other side, female nurses are unable to correctly interpret clients’ instructions, have emotional reactions, lack confidence, are too argumentative in a clinical setting, don’t communicate the procedure before implementation, lack listening skills, are disinterested in patient’s needs, are impatient, not responding to clients’ needs, delay in responding to patient demand, inefficient, less attentive, lack caring spirit, possess anger, doing tasks hastily like having other things to attend, and appeared visibly stressed. All negative qualities from both genders have negative repercussions on patient care, there is a need for efforts to eradicate these negative qualities from both genders. Meanwhile, the negative qualities of male nurses affect clients during medication adherence, affect physicians in their practices, increase clients’ frustration, and scared, erode clients’ trust in the healthcare system, make clients hesitate to seek medical help, and leave clients feeling uncomfortable. For negative qualities of female nurses, they cause clients to feel neglected and burdened, and erode clients’ trust in the healthcare system. It has been found that regardless of gender, the negative qualities abrade patient’s trust in the healthcare system. The negative effects are more common to clients than colleagues.

### ④ positive value of male nurses in clinical facilities from colleagues and clients

Male nurses are reported to be more valued by clients and their colleagues. They are appreciated based on the suggestions they contribute and the patient’s recovery. Based on the history of nursing being regarded as a female profession, male nurses might have improved their education in the form of competency and clinical practices to mask the notion of a female-oriented profession and reverse the negative image of nursing by communities. This conforms to the previous documentation showing that ten years ago male nurses entitled as nurse practitioners increased by 108% [47], which can be an indicator of increasing their values.

### ⑤ different cooperation between male and female nurses in the settings

The majority of participants revealed the existing good teamwork between male and female nurses. This can be reflected in the organized patient care that results in patient recovery. The finding is consistent with a previous study that reported a positive correlation between nurses’ teamwork [48]. If male and female nurses don’t collaborate the disintegrated care will be observed and end up with suboptimal care to clients. For some participants who reported the poor interaction of male and female nurses, it may be due to some few nurses who are still led by the nursing history that a certain gender is inefficient for a certain performance and should not be involved.

### ⑥ mixed perspectives towards clinical competencies across nursing gender

Most of the participants reported their negative perspectives toward male and female nurses. Male nurses are considered unable to offer counseling to nursing mothers and female nurses are regarded as unable to emergency issues. All these perspectives are tied to social and cultural gender roles, assuming that men aren’t supposed to care for women with maternity or gynecological issues. The findings is aligned with the previous study indicating that male nurses are incapable or incompetent in providing intimate care, particularly to young female patients, and cannot adequately take care of female patients, as they are unable to control their sexual impulses and are at risk for sexually assaulting young women [49]. Meanwhile, women are not in a position to respond to emergencies like lifting clients in comas and making prompt decisions.

### ⑦ perspective towards gender diversity in nursing

Due to the demand for diversity in nursing, it is emphasized to eradicate anything hindering the growth of diversity. Moreover, most of the participants had the perspective that the performance of nurses is not determined by their gender, but rather by their competencies. This can be because all nurses regardless of gender are academically trained, molded, and equipped through structured curricula. All nurses are well-informed about the values and ethics of nursing and are encouraged to deliver care professionally. Therefore, their performance will always be based on professionalism rather than their gender personal elements. This is consistent with the previous finding reported that nurses of any gender with professional expertise and good virtues matter more than gender [50].

Diversity of nursing gender is reported to have benefits for clients and nurses themselves. It influences the clients to express their needs freely, promotes patient care, improves patient outcomes, saves the patient’s life, allows diverse skills to be utilized during patient care, promotes healthcare providers’ collaboration which is beneficial to clients, addresses the diverse needs of clients, and promote a holistic approach to patient care. Patient freedom is associated with an opportunity that the patient to choose the preferred gender to be cared for. Optimal patient care and good outcomes are the results of different nurse genders with different capabilities and critical thinking involved in assessment, diagnosis, and decision of care. Similar findings has been previously reported that gender diversity improves cultural competence and outcomes for patients [51].

Nevertheless, to nurses, diversity enhances the overall team dynamic, creates a supportive work environment, learn from each other, shares insights, promotes collaboration, enhances inclusive decision-making, influences personal comfort, and ease of communication and coordination. Moreover, gender diversity fosters problem-solving approaches and brings different perspectives. Having a diverse nursing gender may help some to choose who to work with that may promote a convenient environment for them to be comfortable in their performances. Additionally, the diversity of nursing helps nurses to identify multiple characteristics possessed by nurses and learn those that seem effective in nursing and discard undesirable ones. Consistently, the previous study reported that the diversity of nursing gender encourages nurses to learn from each other and appreciate different perspectives and life experiences [52].

### ⑧ preferences of nurses’ gender, reasons, and opinion towards gender preferences

Some participants indicated having no gender preference for nurses and others had a preference for nurses of specific gender. Meanwhile, some had a preference for nurses of the same gender. Their reasons for preferences were; when having personal discussions or sensitive issues, nurse’s competency, kind of needed service, cultural issues, expectation of comfort, trusting the healthcare system, and past experiences in medical issues. Therefore, gender diversity in nursing is inevitable as it accommodates the different needs of clients. It is advised to embrace diversity and create a cultural environment of promotes diversity.

### Study limitation

Even though the study had a sufficient number of FGDs at different hospitals, it was only confined to a single region out of 28 regions in Tanzania. Therefore, the findings lack a representation of participants from different angles of the country. The study also lacks nursing gender perspectives from student nurses, nursing faculty, employers, and policymakers.

## Conclusion

Male nurses and female nurses differ in how they communicate, execute leadership roles, and have positive clinical qualities. However, their variations don’t mean one gender is underrated than the other, but every gender has unique communication styles, leadership styles, and positive clinical qualities that both lead to effective outcomes. Since all styles, approaches, and qualities are beneficial, every nurse needs to possess all of them, and in contrast, since all negative qualities from both genders have negative repercussions on patient care, there is a need for efforts to eradicate these negative qualities from both genders. Diversity in nursing gender is very important and should be strategized as it is essential to patient recovery and promotes teamwork. Preferences of nursing gender should be encouraged because it enhances somebody’s freedom and creates an environment where a person can discuss sensitive issues. The study has introduced uncertainties that call for further quantitative studies to assess several variables of nursing gender.

### The implications of the study

These results build on existing evidence that gender diversity in nursing has a clinical implication as it improves patients care. Both genders in nursing have negative qualities that calls for innovative strategies to combat these negativities. Moreover, the variation in communication and leadership among nursing gender does not affect the delivery of healthcare services.

### Electronic supplementary material

Below is the link to the electronic supplementary material.

Supplementary Material 1

Supplementary Material 2

Supplementary Material 3

Supplementary Material 4

## Data Availability

The supplementary materials including raw data in the analysis are available upon request.

## Abbreviations

FGDs: Focus Group Discussions
FGD-N: Focus Group Discussions of Nurses
FGD-NN-HCP: Focus Group Discussions of non-nurse healthcare providers
FGD1-C: Focus Group Discussions of clients
DNP: Doctor of Nursing Practice
TNMC: Tanzania Nursing and Midwifery Council
TCU: Tanzania Commission for Universities
MNH: Muhimbili National Hospital
IRREC: Institution Research Review Committee
RAS: Regional Administrative Secretary
NN-HCPs: Non-nurse healthcare providers
