# Supplementary material for: Nurses, non-nurse healthcare providers, and clients’ perspectives, encounters, and choices of nursing gender in Tanzania: a qualitative descriptive study
Source: BMC Nurs. 2024 May 27;23:353. doi: 10.1186/s12912-024-02027-3 (PMC11129494; doi:10.1186/s12912-024-02027-3)
Supplement: Supplementary file 1 — Supplementary Material 1 [file 12912_2024_2027_MOESM1_ESM.doc]

**Supplementary Data 1: Interview guide for nurses, non-nurse healthcare providers, and clients regarding their perspectives, encounters and choices of nursing gender in Tanzania**

1. What is your perspective of nursing gender in Tanzania?

*Probe I: What is your perspective based on experience*

*Probe 2: What is your perspective based on nursing history*

*Probe 3: What is your perspective based on what other people say?*

1. What is your opinion about nursing gender in Tanzania?

*Probe I: What is your opinion about nursing gender to the nursing profession?*

*Probe II: What is your suggestion about nursing gender to the healthcare facilities?*

*Probe III: What is your opinion about nursing gender to the public?*

1. What can you say about your preference for nursing gender when you are seeking medical attention?

*Probe I: What is your preference of nursing gender when seeking medical attention?*

*Probe II: What is your opinion about public preference when seeking medical attention?*

**Common probing questions**

- Can you explain more about what you have just said?
- Can you give an example of what you have mentioned?
- Do you think there are still other issues you want to speak about?
- Has anything important been left out or forgotten that you would like to share?
