# Supplementary material for: Nurses, non-nurse healthcare providers, and clients’ perspectives, encounters, and choices of nursing gender in Tanzania: a qualitative descriptive study
Source: BMC Nurs. 2024 May 27;23:353. doi: 10.1186/s12912-024-02027-3 (PMC11129494; doi:10.1186/s12912-024-02027-3)
Supplement: Supplementary file 2 — Supplementary Material 2 [file 12912_2024_2027_MOESM2_ESM.docx]

**Supplementary Data 2: Analysis of nurse’s perspectives, encounters, and choice on gender nursing in Tanzania**

- **Relational content analysis** has been used in the current analysis to identify concepts in content by finding the relationships between the concepts. **Proximity analysis** being a subcategory of relational content analysis helped to analyze the relationship between concepts and derive a concept matrix from which meanings were developed.
- Regarding coding*,* **In Vivo Coding** has been utilized using participant’s own words to stay as close to their intent and meaning as possible.
- The analysis is done through study objectives.

1. **Objective I: To examine nurse’s perspectives on gender among nurses and nursing practice in Tanzania.**

| **Meaning Unit** | Condensed meaning unit (Description close to the text) | **Subthemes** | **Themes** |
| --- | --- | --- | --- |
| - Communication styles can vary between male and female. - Individual personalities and communication skills influence communication than strict gender differences. - certain skills were characteristically better performed by male nurses | - Communication styles can vary between male and female. | Variation in communication between male nurses and female nurses | Variations of male and female nurses in communication and clinical skills |
|  | - Individual personalities and communication skills influence communication than strict gender differences. - individual personalities and experiences shaping communication preferences | Factor influencing communication for male and female nurses |  |
|  | - certain skills were characteristically better performed by male nurses | Difference in skills between male and female nurses |  |
|  |  |  |  |
| - Collaboration - nurse’s expertise - calm character - empathetic approach - attention to details - skill for analyzing data - skill for recognizing any changes in the patient's condition - more direct - authoritative - tend to be more direct and concise in their communication - more straightforward - solution-oriented communication style - Her calm demeanor - effective communication - calmly coordinated the team in a precise and good way - more empathetic - nurturing - more empathetic in communication - detailed in their communication. - focus on emotional aspects during communication - she had misinterpreted patients the patients’ instruction in regard to a prescribed drug - she was so angry for no reason - inappropriate comments about my uniform - ability to connect with young patients and their families - adaptability in nursing - effective communication - Female healthcare providers, especially nurses, often excel in building rapport and fostering open communication - female nurses are sometimes assumed to be less assertive or confident, especially in decision-making situations - innovative care strategies | - Good collaboration - effective communication | Both male and female nurses’ positive qualities in clinical practice | Mixed clinical qualities across nursing gender |
|  | - nurse’s expertise - calm character - empathetic approach - attention to details - skill for analyzing data - skill for recognizing any changes in the patient's condition - more direct - tend to be more direct and concise in their communication - more straightforward - solution-oriented communication style - ability to connect with young patients and their families. - adaptability in nursing | Male nurses’ positive qualities in clinical practice |  |
|  | - Authoritative - inappropriate comments about my uniform | Male nurses’ negative qualities in clinical practice |  |
|  | - Her calm demeanor - effective communication - calmly coordinated the team in a precise and good way - more empathetic - nurturing - more empathetic in communication - Detailed in their communication. - Focus on emotional aspects during communication. - Female healthcare providers, especially nurses, often excel in building rapport and fostering open communication. - innovative care strategies | Female nurses’ positive qualities in clinical practice |  |
|  | - she had misinterpreted patients the patients’ instruction in regard to a prescribed drug - she was so angry for no reason - female nurses are sometimes assumed to be less assertive or confident, especially in decision-making situations | Female nurses’ negative qualities in clinical practice |  |
|  |  |  |  |
| - put all of us at ease - created a sense of trust - mutual respect - accuracy of our assessments and interventions. - team dynamics | - put all of us at ease - created a sense of trust - mutual respect - Accuracy of our assessments and interventions. | Positive effect of male nurse qualities in clinical setting | The positive effects of male and female nurse’s qualities in a clinical setting |
|  | - team dynamics | Positive effect of female nurse qualities in clinical setting |  |
|  |  |  |  |
| - fellow male nurse colleagues received more immediate trust and respect from patients - if the suggestion come from a male colleague who is a nurse it is accepted - male colleagues were given more credit for successful patient outcomes - male colleagues received more immediate acknowledgment | - if the suggestion come from a male colleague who is a nurse it is accepted. - male colleagues were given more credit for successful patient outcomes - male colleagues received more immediate acknowledgment | Value of male nurses by colleague | The positive value of male nurses in a clinical facilities from colleague and patients |
|  | - fellow male nurse colleagues received more immediate trust and respect from patients | Value of male nurses from patients |  |
|  |  |  |  |
| - me that I was also a mere male nurse and should not bossy her - our communication was strained - it affected the overall coordination of care | - me that I was also a mere male nurse and should not bossy her - our communication was strained - it affected the overall coordination of care | Relationship of male nurses and female nurses in clinical practice | Interaction of male and female nurses at clinical practices |
|  |  |  |  |
| - my ward in charge assumes that I can’t offer counselling specially to nursing mothers simply because of my gender - openly asked if I know how to display empathy - a patient assumed I was less knowledgeable simply because I'm a male nurse. - she was assumed to be less capable of handling emergency situations simply because of her gender and a male nurse in charge of a particular unit was tasked to handle the situation. - Despite the female nurse being experienced and skilled, there was an automatic assumption that a male nurse would handle critical cases better. - male nurses, assuming they had a better understanding of the clients’ nursing aspects. - patients assuming that male healthcare providers even our fellow nurses, hold more authority automatically | - my ward in charge assumes that I can’t offer counseling specially to nursing mothers simply because of my gender - openly asked if I know how to display empathy | Negative perception towards male nurses’ clinical competency | Negative perceptions towards clinical competencies across nursing gender |
|  | - she was assumed to be less capable of handling emergency situations simply because of her gender and a male nurse in charge of a particular unit was tasked to handle the situation. - Despite the female nurse being experienced and skilled, there was an automatic assumption that a male nurse would handle critical cases better. | Negative perception towards female nurses’ clinical competency |  |
|  |  |  |  |
| - patients sometimes feel more comfortable discussing certain concerns with healthcare providers of the same gender - may contribute to a more comfortable experience. - the patient feel comfortable - supportive environment - patients' preferences are respected - Having a team with a variety of genders allows patients to have choices - can help the team adapt and ensure that every patient receives personalized and respectful care - improving patient outcomes - creates a collaborative environment that directly benefits patient care. - better outcomes for patients. - diverse skills and perspectives are utilized for the benefit of the patient - friendship enhances the overall team dynamic - has created a supportive work environment - We learn from each other - share insights - collaboration - inclusive decision-making - everyone's input matters - inclusive nursing culture - personal comfort - ease of communication - a supportive team environment promotes effective communication and coordination - a mix of male and female nurses brings different perspectives - problem-solving approaches. - saving the patients life. - crucial for providing patient-centered care. - Creating a safe space for patients to express their needs and ensuring that those preferences are honored. - Contribute to a more understanding and inclusive environment. - overall patient experience - well-rounded patient care. - created an enjoyable work environment | - patients sometimes feel more comfortable discussing certain concerns with healthcare providers of the same gender - patients' preferences are respected - creating a safe space for patients to express their needs and ensuring that those preferences are honored - Having a team with a variety of genders allows patients to have choices - may contribute to a more comfortable experience. - supportive environment - overall patient experience | Importance of nursing gender diversity to patient’s freedom | Distinct importance of nursing gender diversity to patients and nurses |
|  | - well-rounded patient care. - creates a collaborative environment that directly benefits patient care. - diverse skills and perspectives are utilized for the benefit of the patient - crucial for providing patient-centered care - the patient feel comfortable - can help the team adapt and ensure that every patient receives personalized and respectful care - improving patient outcomes - saving the patients life. | Importance of nursing gender diversity to patient’s care and outcome |  |
|  | - friendship enhances the overall team dynamic - has created a supportive work environment - We learn from each other - share insights - collaboration - inclusive decision-making - everyone's input matters - inclusive nursing culture - personal comfort - ease of communication - a supportive team environment promotes effective communication and coordination - a mix of male and female nurses brings different perspectives - Problem-solving approaches. - Contribute to a more understanding and inclusive environment. - created an enjoyable work environment | Importance of nursing gender diversity to nurses |  |
|  |  |  |  |
| - need for continuous efforts to challenge and overcome gender biases in healthcare - the need for eliminate gender-based assumptions - emphasizes the importance of recognizing and addressing biases to ensure that every nurse, irrespective of gender, has the opportunity to contribute fully to patient care. - values diversity - embracing diversity as a strength within the team. - creating a diverse and inclusive healthcare environment is important. - I believe that fostering a culture that values diversity and inclusivity within the nursing team can contribute to breaking down gender biases. - it's always been about the nurse's competence and ability to provide effective care. - I believe competence and professionalism are crucial in healthcare, regardless of gender. - the need for nurses to be evaluated based on their capabilities rather than predefined gender roles - the need for a shift towards recognizing and valuing competence and skills over traditional gender roles. - skills and competence are valued regardless of gender - nursing excellence is not bound by gender - Also, providing equal opportunities for professional development and advancement can contribute to a more balanced distribution of roles. - ensuring fair treatment and opportunities for everyone. - Providers should encourage patients to express their preferences and concerns regarding gender - should encourage patients to express their preferences and concerns, including those related to gender. - treats every nurse with respect and fairness - fostering a culture of collaboration - shared responsibility. - creating an open and non-judgmental environment where patients feel comfortable expressing their preferences. - everyone's strengths were recognized and utilized, regardless of gender. - competence, empathy, and effective communication are essential qualities, irrespective of gender. - importance of mutual respect and recognition of expertise, irrespective of gender. - qualities like empathy and adaptability are crucial in healthcare, and they are not bound by gender. - nursing is about dedication. - effective nursing care is not limited by gender. - collaboration and a shared commitment to patient well-being. - establish a welcoming environment where patients feel comfortable expressing their preferences. - healthcare providers can initiate conversations with patients about their preferences - open communication is also important whereby we create an environment where patients feel comfortable expressing their preferences - nursing excellence is not bound by gender. | - values diversity - Embracing diversity as a strength within the team. - creating a diverse and inclusive healthcare environment is important. | The need for diversity in nursing | Perception towards gender diversity in nursing |
|  | - need for continuous efforts to challenge and overcome gender biases in healthcare - the need for eliminate gender-based assumptions - emphasizes the importance of recognizing and addressing biases to ensure that every nurse, irrespective of gender, has the opportunity to contribute fully to patient care - Allows nurses to address any concerns related to gender or other factors that might impact patient care. | The need to address gender biases in nursing |  |
|  | - it's always been about the nurse's competence and ability to provide effective care. - I believe competence and professionalism are crucial in healthcare, regardless of gender. - the need for nurses to be evaluated based on their capabilities rather than predefined gender roles - the need for a shift towards recognizing and valuing competence and skills over traditional gender roles. - skills and competence are valued regardless of gender - nursing excellence is not bound by gender - everyone's strengths were recognized and utilized, regardless of gender. - competence, empathy, and effective communication are essential qualities, irrespective of gender. - importance of mutual respect and recognition of expertise, irrespective of gender. - qualities like empathy and adaptability are crucial in healthcare, and they are not bound by gender. - nursing is about dedication. - effective nursing care is not limited by gender. - collaboration and a shared commitment to patient well-being. - nursing excellence is not bound by gender. | The relation of nursing gender and competence |  |
|  |  |  |  |
|  |  |  |  |
| - having a preference, especially in certain situations where discussing sensitive issues is involved - I haven't had a specific preference for a nurse's gender. - I also have never had any preference for a nurse's gender. - I found that having a female nurse created a more comfortable environment for me to openly communicate about personal health matters. - being a male nurse, I have in some situations, felt more comfortable discussing personal health issues with a female nurse - female patients who explicitly requested a female nurse for intimate procedures - she was undergoing and some discussions about personal health matters - I look for and want is competence, communication skills, and ability to provide compassionate care. - The most important factors for me are their professionalism and expertise - feels more comfortable with someone of the same gender. - female patient specifically requested a female nurse for a sensitive procedure - The female patient expressed discomfort with a male nurse due to personal reasons. - Patients seem to feel more at ease sharing their concerns and asking questions with female providers. - some patients, influenced by cultural factors, - I expect competence and professionalism of the provider regardless to gender - sometimes I seek out people purely for personal reasons. - even if I am a male nurse, I feel more comfortable discussing personal health issues with a female nurse. | - even if I am a male nurse, I feel more comfortable discussing personal health issues with a female nurse. | Preferring nurse of opposite gender | Preferences of nurse’s gender, reasons, opinion, and challenges towards gender preferences |
|  | - female patients who explicitly requested a female nurse for intimate procedures - female patient specifically requested a female nurse for a sensitive procedure - I've felt more at ease with healthcare providers of the same gender. | Preferring nurse of same gender |  |
|  | - I haven't had a specific preference for a nurse's gender. - I also have never had any preference for a nurse's gender. | Having no gender preference for nurses |  |
|  | - having a preference, especially in certain situations where discussing sensitive issues is involved - I found that having a female nurse created a more comfortable environment for me to openly communicate about personal health matters. - being a male nurse, I have in some situations, felt more comfortable discussing personal health issues with a female nurse - The most important factors for me are their professionalism and expertise in delivering quality healthcare. - she was undergoing and some discussions about personal health matters - I look for and want is competence, communication skills, and ability to provide compassionate care. - The most important factors for me are their professionalism and expertise in delivering quality healthcare. - feels more comfortable with someone of the same gender. - The female patient expressed discomfort with a male nurse due to personal reasons. - Patients seem to feel more at ease sharing their concerns and asking questions with female providers. - some patients, influenced by cultural factors, - I expect competence and professionalism of the provider regardless to gender - sometimes I seek out people purely for personal reasons. | Reasons for gender preferences |  |
| - Providers should encourage patients to express their preferences and concerns regarding gender - should encourage patients to express their preferences and concerns, including those related to gender. - establish a welcoming environment where patients feel comfortable expressing their preferences. - healthcare providers can initiate conversations with patients about their preferences - open communication is also important whereby we create an environment where patients feel comfortable expressing their preferences | - Providers should encourage patients to express their preferences and concerns regarding gender - Should encourage patients to express their preferences and concerns, including those related to gender. - Establish a welcoming environment where patients feel comfortable expressing their preferences. - healthcare providers can initiate conversations with patients about their preferences - open communication is also important whereby we create an environment where patients feel comfortable expressing their preferences | Opinion about gender preferences |  |
| - hectic day and few staff available   fail to attain our goal. | - hectic day and few staff available   fail to attain our goal | Challenge of meeting patient’s preferences through nurse’s gender diversity |  |
|  |  |  |  |
