# Supplementary material for: Nurses, non-nurse healthcare providers, and clients’ perspectives, encounters, and choices of nursing gender in Tanzania: a qualitative descriptive study
Source: BMC Nurs. 2024 May 27;23:353. doi: 10.1186/s12912-024-02027-3 (PMC11129494; doi:10.1186/s12912-024-02027-3)
Supplement: Supplementary file 3 — Supplementary Material 3 [file 12912_2024_2027_MOESM3_ESM.docx]

**Supplementary Data 3: Analysis of HCP’s perspectives, encounters, and choices on gender nursing in Tanzania**

- **Relational content analysis** has been used in the current analysis to identify concepts in content by finding the relationships between the concepts. **Proximity analysis** being a subcategory of relational content analysis helped to analyze the relationship between concepts and derive a concept matrix from which meanings were developed.
- Regarding coding*,* **In Vivo Coding** has been utilized using participant’s own words to stay as close to their intent and meaning as possible.
- The analysis is done through study objectives.

**(ii) Objective I: To explore HCP’s perspectives on gender among nurses and nursing practice in Tanzania.**

| **Code** | **Categories** | **Subthemes** | **Themes** |
| --- | --- | --- | --- |
| - Collaboration - female nurses seemed more comfortable discussing sensitive patient issues with female doctors - female nurses feel more comfortable discussing patient concerns or sharing critical information with female doctors | - There is Collaboration | Status of collaboration between nurses and HCP | Interaction of HCP with nurses and between female and male nurses at clinical setting  . |
|  | - Female nurses seemed more comfortable discussing sensitive patient issues with female doctors. - female nurses feel more comfortable discussing patient concerns or sharing critical information with female doctors | Female nurses’ collaboration preference |  |
| - Their ability to work together, leveraging individual strengths, - Nurses collective efforts in providing care showcase their professionalism and dedication - incredible teamwork among nurses - Their unity in providing patient-centered care - Collaboration among nurses | - Their ability to work together, leveraging individual strengths. - Nurses’ collective efforts in providing care showcase their professionalism and dedication. - incredible teamwork among nurses - Their unity in providing patient-centered care. - Collaboration among nurses | Interaction of male and female nurses at clinical practices |  |
| - accurate test results. - maintaining a smooth workflow - delivering high-quality patient care. | - Accurate test results. - maintaining a smooth workflow - Delivering high-quality patient care. | Impact of collaboration of HCP and nurses |  |
| - differences in the communication styles between male and female nurses when conveying information about patient medications - There's a subtle difference in how they approach discussions about drug interactions or side effects - I've noticed that there might be variations in how male and female colleagues express their opinions or share information during team discussions - individual communication styles and personalities rather than strictly from gender. - male nurses have been more concise - female nurses might provide more contextual information about the patient's condition. - differences in how male and female nurses communicate prescription details or inquire about medication-related issues - enrich the overall quality of care and everyone has his or her own flavor - the variations in communication styles among male and female nurses that I might have notices is when they are discussing medication-related matters - Female nurses give rather long and detailed approach - male nurses might are a little birief - There's a difference in how male and female nurses direct these conversations and I have seen that patients respond differently based on the gender of the nurse leading the discussion. - how male and female nurses approach seeking clarification or conveying information regarding prescriptions or medication-related questions - male nurses approach communication in a rather more direct | - There's a difference in how male and female nurses direct these conversations and I have seen that patients respond differently based on the gender of the nurse leading the discussion. - Differences in the communication styles between male and female nurses when conveying information about patient medications. - There's a subtle difference in how they approach discussions about drug interactions or side effects. - there's a difference in how male and female nurses approach discussing drug interactions or side effects with patients. - how male and female nurses conveying information regarding prescriptions or medication-related questions | Different in communication of male and female nurses when conveying information to patients | Variations of male and female nurses in communication |
|  | - I've noticed that there might be variations in how male and female colleagues express their opinions or share information during team discussions. - differences in how male and female nurses communicate prescription details or inquire about medication-related issues. - there's a difference in how male and female nurses approach discussing drug interactions even communicating with us - how male and female nurses approach seeking clarification regarding prescriptions or medication-related questions | Different in communication of male and female nurses when discussing with team members |  |
|  | - the variations in communication styles among male and female nurses that I might have notices is when they are discussing medication-related matters | Different in communication of male and female nurses when discussing about medication related issues |  |
|  | - male nurses have been more concise - male nurses are little brief - male nurse might come in with a prescription and request for drugs and leave - male nurses approach communication in a rather more direct - male nurses might focus more on factual information - male nurses tend to be assertive in their communication during critical situations | Communication approaches by male nurses |  |
|  | - female nurses might provide more contextual information about the patient's condition. - Female nurses give rather long and detailed approach - female nurse might ask some specific details as in what type of food the patient should be told to eat and other basic stuff in regard to the drugs - female nurses tend to be more empathetic and nurturing - They excel in building rapport and establishing trust | Communication approaches by female nurses |  |
|  | - individual communication styles and personalities rather than strictly from gender. - the gender of the nurse can influence how information is conveyed. - communication or collaboration haven't been gender-specific; they were more individual-based. | Factor influencing communication approaches for male and female nurses |  |
|  | - enrich the overall quality of care and everyone has his or her own flavor | Impact of difference in communication of male and female nurses |  |
| - I have also noticed some slight variation in leadership styles between male and female nurses. - varied leadership styles among male and female nurses. | - I have also noticed some slight variation in leadership styles between male and female nurses. - female nurses exhibit more collaborative leadership. - male nurses might take a more authoritative approach | Variation in leadership across nursing gender | Variations of male and female nurses in leadership |
| - empathy, communication, and teamwork in nursing care - show a remarkable ability to work together cohesively. - Collaboration - shared commitment to patient well-being - male nurses tend to be more direct and assertive in their communication during critical situations - male nurses being more direct - taking a more collaborative approach. - Male nurses, on the other hand, can bring a sense of calm and confidence, especially in high-pressure situations. - some male colleagues who portray the stated empathetic approach - some female nurses might approach the same scenarios with a more empathetic or nurturing manner. - female nurses assign tasks or seek assistance - female nurses often excel in creating a compassionate and empathetic environment for patients. - Female colleagues often bring a more empathetic and nurturing approach - male nurses appeared more confident in voicing their opinions or suggestions during patient rounds - female healthcare providers and more so nurses often excel in building rapport and establishing trust with patients, emphasizing collaboration and a holistic approach to care unlike male nurses - female nurses I work with often prioritize team collaboration and open communication - male and female nurses excel in their roles - female nurses tend to excel in creating a comfortable and supportive environment for patients - male nurses although male nurses are kind of trusted more - nurses' dedication to delivering high-quality care remains consistent regardless of gender identity - demonstrate remarkable teamwork and dedication to patient care. - Assertive - female nurse they often prioritize collaboration - male nurses sometimes adopt directive approach in team settings. - male nurses who are more compassionate - male nurses are as empathetic - appreciate the diverse skill sets and approaches that both male and female nurses bring to patient care. - women tend to be more nurturing or compassionate - Majority of male nurses would love to be addressed as doctors by patients and they do want to assume they are doctors and forget their roles as nurses who have specified roles and responsibilities. - male nurses as people who one has to be careful with as they have the tendency of spreading rumors - male nurses are kind of like a lot of “fitina” - male nurses they show a more task-oriented approach, focusing on efficient execution of care plans | - empathy, communication - teamwork in nursing care - show a remarkable ability to work together cohesively. - shared commitment to patient well-being - male and female nurses excel in their roles - nurses' dedication to delivering high-quality care remains consistent regardless of gender identity - appreciate the diverse skill sets and approaches that both male and female nurses bring to patient care. - appreciate the strengths each nurse possesses | Both male and female nurses’ positive qualities in clinical practice | Divergent clinical qualities across nursing gender |
|  | - male nurses being more direct - taking a more collaborative approach. - Male nurses, on the other hand, can bring a sense of calm and confidence, especially in high-pressure situations. - some male colleagues who portray the stated empathetic approach - male nurses appeared more confident in voicing their opinions or suggestions during patient rounds. - male nurses are more of what does the rules state - too much of protocol - male nurses are kind of more trusted - male nurses sometimes adopt directive approach in team settings. - male nurses who are more compassionate - male nurses are as empathetic - male nurses they show a more task-oriented approach, focusing on efficient execution of care plans | Male nurses’ positive qualities in clinical practice |  |
|  | - Assertive - Majority of male nurses would love to be addressed as doctors by patients and they do want to assume they are doctors and forget their roles as nurses who have specified roles and responsibilities. - male nurses as people who one has to be careful with as they have the tendency of spreading rumors - male nurses are kind of like a lot of “fitina” | Male nurses’ negative qualities in clinical practice |  |
|  | - some female nurses might approach the same scenarios with a more empathetic or nurturing manner. - female nurses assign tasks. - seek assistance. - female nurses often excel in creating a compassionate and empathetic environment for patients. - Female healthcare providers and more so nurses often excel in building rapport and establishing trust with patients, emphasizing collaboration and a holistic approach to care unlike male nurses. - female nurses work with often prioritize team collaboration and open communication. - female nurses tend to excel in creating a comfortable and supportive environment for patients - some female nurses being seen as more understanding. - fostering open communication | Female nurses’ positive qualities in clinical practice |  |
|  | - too argumentative | Female nurses’ negative qualities in clinical practice |  |
|  |  |  |  |
| - affect the overall collaboration - patients to respond differently to medication adherence discussion - Male nurses accepting to be considered as doctors hampers my work | - affect the overall collaboration | Positive effect of male nurse qualities in clinical setting | Positive and negative effects of male and female nurse’s qualities in clinical setting |
|  | - patients to respond differently to medication adherence discussion. - Male nurses accepting to be considered as doctors hampers my work | Negative effect of male nurse qualities in clinical setting |  |
|  | - influencing how patients engage in these conversations. | Positive effect of female nurse qualities in clinical setting |  |
|  |  |  |  |
| - that male nurses sometimes encounter assumptions about their capabilities from patients | - male nurses sometimes encounter assumptions about their capabilities from patients | Negative perception towards male nurses’ clinical competency | Patients’ negative perceptions towards male nurses’ clinical competencies |
|  |  |  |  |
| - varied perspectives and communication styles contribute significantly to patient care. - contribute to a well-rounded and patient-focused care environment. - gender mix among nurses can enhance the overall team dynamic. - better teamwork and understanding. - enriching our overall healthcare delivery - ability to connect with patients - quality care - collaborate within healthcare teams. - address the diverse needs of patients. - diversity among nurses enhances the collaborative nature of healthcare teams - bring unique perspectives and strengths - holistic approach in patient care. - enriches nursing practice - brings valuable perspectives - influence team dynamics among nurses | - varied perspectives and communication styles contribute significantly to patient care. - contribute to a well-rounded and patient-focused care environment. - address the diverse needs of patients. - impact patient care - holistic approach in patient care. | Importance of nursing gender diversity to patient’s care | Distinct importance of nursing gender diversity to patients and nurses |
|  | - fosters an environment conducive to excellent patient outcomes. | Importance of nursing gender diversity to patient’s outcome |  |
|  | - gender mix among nurses can enhance the overall team dynamic. - better teamwork and understanding. - enriching our overall healthcare delivery - ability to connect with patients. - quality care - diversity among nurses enhances the collaborative nature of healthcare teams. - bring unique perspectives and strengths. - enriches nursing practice. - brings valuable perspectives. - genders bring diverse skill sets and communication styles to their practice. | Importance of nursing gender diversity to nurses |  |
|  |  |  |  |
| - importance of a gender-diverse nursing workforce. - appreciate the diverse perspectives and approaches - The gender diversity within nursing should be celebrated - expectations should be based on individual capabilities and skills rather than gender stereotypes - valuing individual strengths and skills in nursing rather than attributing them solely to gender. - cknowledging and valuing diverse communication styles regardless to gender - the importance of embracing and respecting diverse styles - appreciate the diverse strengths of nurses - I expect a high level of professionalism and dedication from nursing personnel focusing more on competency than gender-specific traits - that gender doesn't determine competency but rather contributes to a rich diversity in nursing practice. - appreciate individual strengths and communication styles - nursing is a field where competence and compassion matter more than gender - expect professionalism and dedication from nurses, irrespective of gender. - understand the importance of individual strengths and skills rather than gender in nursing. - emphasized the importance of their diverse skill sets | - importance of a gender-diverse nursing workforce. - appreciate the diverse perspectives and approaches - The gender diversity within nursing should be celebrated - acknowledging and valuing diverse communication styles regardless to gender - the importance of embracing and respecting diverse styles - appreciate the diverse strengths of nurses - appreciate individual strengths and communication styles - emphasized the importance of their diverse skill sets - diverse approaches are valued | The need for diversity in nursing | Perception towards gender diversity |
|  | - expectations should be based on individual capabilities and skills rather than gender stereotypes. - valuing individual strengths and skills in nursing rather than attributing them solely to gender. - qualities like compassion, expertise, and effective collaboration, understanding that these attributes aren't tied to any specific gender. - acknowledge individual skills and communication styles rather than categorizing based on gender. - I expect a high level of professionalism and dedication from nursing personnel focusing more on competency than gender-specific traits. - that gender doesn't determine competency but rather contributes to a rich diversity in nursing practice. - nursing is a field where competence and compassion matter more than gender. - expect professionalism and dedication from nurses, irrespective of gender. - understand the importance of individual strengths and skills rather than gender in nursing. | The nurse’s competency is not bound by gender |  |
| - I've encountered patients who had preferences for a nurse of a specific gender while receiving instructions or explanations about their medications. - preferences are often linked to personal comfort levels and the nature of the information being shared. - some female patients might feel more comfortable discussing certain personal or sensitive issues with female doctors or nurses - personal comfort - cultural considerations. - patients may feel more at ease and communicate more openly with a nurse of the same gender. - They've mentioned patients feeling more at ease or open to discussing certain health issues with nurses of their preferred gender | - I've encountered patients who had preferences for a nurse of a specific gender while receiving instructions or explanations about their medications. - They've mentioned patients feeling more at ease or open to discussing certain health issues with nurses of their preferred gender. - felt more at ease discussing sensitive topics like personal hygiene or reproductive health with a nurse of the same gender | Having preference of nurse of specific gender | Status, reasons,challenges, and opinion towards gender preferences |
|  | - some female patients might feel more comfortable discussing certain personal or sensitive issues with female doctors or nurses - patients may feel more at ease and communicate more openly with a nurse of the same gender. - preference for same-gender care providers during intimate procedures or examinations | Having preference of nurse of same gender |  |
|  | - patients didn’t have a preference and were comfortable with healthcare providers of any gender. | Having no gender preference for nurses |  |
|  | - preferences are often linked to personal comfort levels - the nature of the information being shared. - cultural considerations. - personal reasons - may trust the person - individual's patient’s personality - past experiences where he or she was either blamed for choosing a provider. - Some patients trust in the healthcare system - due to modesty or religious reasons - patients might feel more at ease | Reasons for gender preferences |  |
| - the scarcity of the workforce - shortage of nurses of that gender, | - the scarcity of the workforce - shortage of nurses of that gender, | Challenge of meeting patient’s preferences through nurse’s gender diversity |  |
| - having open discussions with patients, understanding their preferences - involve open discussions with patients to understand their comfort levels and preferences - listening to patients' preferences - create an environment where patients feel comfortable expressing their preferences without judgment - creating a supportive and non-judgmental space for patients to express their preferences. - acknowledge and respect patient preferences - felt more at ease discussing sensitivetopics like personal hygiene or reproductive health with a nurse of the same gender - personal reasons - may trust the person - individual's patient’s personality - past experienceswhere he or she was either blamed for choosing a provider. - Some patients trust in the healthcare system - Respecting these preferences has been important in maintaining patient trust. | - having open discussions with patients, understanding their preferences - involve open discussions with patients to understand their comfort levels and preferences - listening to patients' preferences - create an environment where patients feel comfortable expressing their preferences without judgment - creating a supportive and non-judgmental space for patients to express their preferences. - acknowledge and respect patient preferences | Opinion about gender preferences |  |
