# Supplementary material for: Nurses, non-nurse healthcare providers, and clients’ perspectives, encounters, and choices of nursing gender in Tanzania: a qualitative descriptive study
Source: BMC Nurs. 2024 May 27;23:353. doi: 10.1186/s12912-024-02027-3 (PMC11129494; doi:10.1186/s12912-024-02027-3)
Supplement: Supplementary file 4 — Supplementary Material 4 [file 12912_2024_2027_MOESM4_ESM.docx]

**Supplementary Data 4: Analysis of Client’s perspectives, encounters, and choices on gender nursing in Tanzania**

- **Relational content analysis** has been used in the current analysis to identify concepts in content by finding the relationships between the concepts. **Proximity analysis** being a subcategory of relational content analysis helped to analyze the relationship between concepts and derive a concept matrix from which meanings were developed.
- Regarding coding*,* **In Vivo Coding** has been utilized using participant’s own words to stay as close to their intent and meaning as possible.
- The analysis is done through study objectives.

**(iii) Objective I: To examine Client’s perspectives on gender among nurses and nursing practice in Tanzania.**

| **Code** | **Categories** | **Subthemes** | **Themes** |
| --- | --- | --- | --- |
| - might be variations in communication styles and approaches for male and female providers - indirect differences in communication styles to empathy among male and female nurses | - might be variations in communication styles and approaches for male and female providers - indirect differences in communication styles to empathy among male and female nurses | Variation in communication between male nurses and female nurses | Variations of male and female nurses in communication and clinical skills |
| - The nurses, both male and female, were generally more attentive and communicative than the doctor in that particular instance. - show qualities like sympathy and communication - I was treated very bad by some nurses - One male nurse was incredibly attentive - and helped me when attending my hospital visit - he answers all my concerns and explains my treatment plan clearly in a way that I feel so well understood and heard. - the nurse displayed practical skillduring a challenging procedure, - he also maintained a kind and comforting approach - ability to effectively communicate - some male nurses were incredibly competent - male nurse who when I arrived he received me and happiliy introduced himself of which I later learned that he was a student. - But the way he handled me and explaining every procedure - gave me the opportunity to ask questions if I had any - I was being cleaned my wound by a male nurse who regardless to telling him I am in severe pain when he was removing the bandages, he did not listen and pulled them painfully and I ended up enduring the pain. - he cleaned the wound very very roughly without giving me and medication to help and after he had finished cleaning, he told me to pick up the used bandages and dispose them myself. - lack of sympathy - male nurses being less attentive and - the lack of empathy and communication from some of the male nurses makes me more conscious of these aspects. - I think that’s why a lot of male nurses in the hospital prefer to be called doctors instead of nurses by patients. - He was brusque in his mannerisms, - displaying a lack of empathy - displaying a lack of concern for my well-being. - he brushed it off, saying, 'It's not that bad, you'll manage - lack of professionalism in maintaining my privacy - more authoritative. - male nurse since all the time I had been referring to him as a doctor. Weeeeeeeeee……. I have never seen that anger, he thought I was making fun of him, threw on the bed my drug and told me to medicate myself. - Sympathetic - She was exceptionally skilled - Her communication was clear - took the time to listen to my concerns - she actively involved me in decisions about my care - Her approach was remarkable - her ability to connect with me on a personal level - her empathetic - compassionate behaviour - female nurses are more caring - Maintain privacy - peacefulness - She not only explained to me my sickness at the time she also took time to thoroughly explain what I was supposed to eat and even offered to direct me to a seller who sells the food. - female nurse that left me extremely uncomfortable - the nurse really appeared visibly stressed and overwhelmed - She didn't announce herself when attending to me just ordered me to turn over and injected me not telling me what she had injected me with or whatever - she rebukes me - and seemed in a rush to attend to other tasks. - she interrupted me multiple times - impatient and seemed disinterested. - she brushed them off - she seemed annoyed - didn't take the time to understand my situation - she administered medication without explaining what it was for or how it would help and without ass - few female nurses seemed inefficient and less attentive - female nurse who seemed like she did not care of my wellbeing - she angrily brushed me off. | - The nurses, both male and female, were generally more attentive and communicative than the doctor in that particular instance. - show qualities like sympathy and communication - I was treated very bad by some nurses | Both male and female nurses’ positive and negative qualities in clinical practice | Divergent clinical qualities across nursing gender |
|  | - One male nurse was incredibly attentive - and helped me when attending my hospital visit - he answers all my concerns and explains my treatment plan clearly in a way that I feel so well understood and heard. - the nurse displayed practical skill during a challenging procedure, - he also maintained a kind and comforting approach - ability to effectively communicate - some male nurses were incredibly competent - male nurse who when I arrived he received me and happiliy introduced himself of which I later learned that he was a student. - But the way he handled me and explaining every procedure - gave me the opportunity to ask questions if I had any | Male nurses’ positive qualities in clinical practice |  |
|  | - I was being cleaned my wound by a male nurse who regardless to telling him I am in severe pain when he was removing the bandages, he did not listen and pulled them painfully and I ended up enduring the pain. - he cleaned the wound very very roughly without giving me and medication to help and after he had finished cleaning, he told me to pick up the used bandages and dispose them myself. - lack of sympathy - male nurses being less attentive and - the lack of empathy and communication from some of the male nurses makes me more conscious of these aspects. - I think that’s why a lot of male nurses in the hospital prefer to be called doctors instead of nurses by patients. - He was brusque in his mannerisms, - displaying a lack of empathy - displaying a lack of concern for my well-being. - he brushed it off, saying, 'It's not that bad, you'll manage - lack of professionalism in maintaining my privacy - more authoritative. - male nurse since all the time I had been referring to him as a doctor. Weeeeeeeeee……. I have never seen that anger, he thought I was making fun of him, threw on the bed my drug and told me to medicate myself. | Male nurses’ negative qualities in clinical practice |  |
|  | - Sympathetic - She was exceptionally skilled - Her communication was clear - took the time to listen to my concerns - she actively involved me in decisions about my care - Her approach was remarkable - her ability to connect with me on a personal level - her empathetic - compassionate behaviour - female nurses are more caring - Maintain privacy - Peacefulness - She not only explained to me my sickness at the time she also took time to thoroughly explain what I was supposed to eat and even offered to direct me to a seller who sells the food. | Female nurses’ positive qualities in clinical practice |  |
|  | - female nurse that left me extremely uncomfortable - the nurse really appeared visibly stressed and overwhelmed - She didn't announce herself when attending to me just ordered me to turn over and injected me not telling me what she had injected me with or whatever - she rebukes me - and seemed in a rush to attend to other tasks. - she interrupted me multiple times - impatient and seemed disinterested. - she brushed them off - she seemed annoyed - didn't take the time to understand my situation - she administered medication without explaining what it was for or how it would help and without ass - few female nurses seemed inefficient and less attentive - female nurse who seemed like she did not care of my wellbeing - she angrily brushed me off. | Female nurses’ negative qualities in clinical practice |  |
| - He removed my anxiety and I felt so peaceful. - I was supposed to be done to made me feeling good. - Frustrating - Scared - It eroded my trust in the healthcare system - nowadays find myself hesitating to seek medical help - I had an unfortunate encounter with a male nurse during my recent hospital stay that left me extremely uncomfortable. - It felt dismissive and insensitive - made me feel vulnerable. - I felt ignored, disrespected, and overall neglected under his care. - making sure I felt informed and involved - made the hospital environment feel more supportive for me. - female nurse who made me feel really comfortable during a sensitive exam - it felt like she wasn't even listening to me. - feel like I was being a burden rather than someone in need of care. - I felt neglected and dismissed. - It eroded my trust in the healthcare system - left me questioning the quality of care provided in such settings. | - He removed my anxiety and I felt so peaceful. - I was supposed to be done to made me feeling good. | Positive effect of male nurse qualities in clinical setting | Effects of male and female nurse’s qualities in clinical setting |
|  | - Frustrating - Scared - It eroded my trust in the healthcare system - nowadays find myself hesitating to seek medical help - I had an unfortunate encounter with a male nurse during my recent hospital stay that left me extremely uncomfortable. - It felt dismissive and insensitive - made me feel vulnerable. - I felt ignored, disrespected, and overall neglected under his care. | Negative effect of male nurse qualities in clinical setting |  |
|  | - making sure I felt informed and involved - made the hospital environment feel more supportive for me. - female nurse who made me feel really comfortable during a sensitive exam | Positive effect of female nurse qualities in clinical setting |  |
|  | - it felt like she wasn't even listening to me. - feel like I was being a burden rather than someone in need of care. - I felt neglected and dismissed. - It eroded my trust in the healthcare system - left me questioning the quality of care provided in such settings. | Negative effect of female nurse qualities in clinical setting |  |
| - They worked together as I was attended by different nurses of morning, afternoon and night shift and they all knew when to give me my medication. I really felt cared for. - unity among nursing - I've witnessed instances where nurses, regardless of gender, have shown great teamwork. - I appreciate the emphasis on teamwork, and it's reassuring to know that the care I receive is a result of collaboration - The collaborative efforts of nurses creates a sense of trust - The collaborative spirit among nurses is something to be stressed on. - I agree that collaborative efforts of nurses create a supportive environment. - Regardless of gender, nurses should work together - the professionalism and unity within the nursing teams | - They worked together as I was attended by different nurses of morning, afternoon and night shift and they all knew when to give me my medication. I really felt cared for. - unity among nursing - I've witnessed instances where nurses, regardless of gender, have shown great teamwork. - I appreciate the emphasis on teamwork, and it's reassuring to know that the care I receive is a result of collaboration | Existing relationship of male nurses and female nurses in clinical practice | Interaction of male and female nurses at clinical practices |
|  | - The collaborative efforts of nurses creates a sense of trust - The collaborative spirit among nurses is something to be stressed on. - I agree that collaborative efforts of nurses create a supportive environment. - Regardless of gender, nurses should work together - the professionalism and unity within the nursing teams | Perception about interaction of male and female nurses at clinical practices |  |
| - male nurse as I know it’s like they were being forced to work in the hospital | - male nurse as I know it’s like they were being forced to work in the hospital. | Negative perception towards male nurses’ clinical competency | Perceptions towards clinical competencies across nursing gender |
| - Lets take the different genders of nursing as a good point as they bring different flavor | - Lets take the different genders of nursing as a good point as they bring different flavor | Importance of nursing gender diversity to nurses |  |
| - one individual's behavior doesn't represent an entire gender or profession - think the hospital need to have both male and female nurses from different parts of Tanzania to bring in different tastes. - both male and female in the nursing profession is an asset. - caring and good communication, are not limited by gender. It's about finding nurses professional - the need for a providing good nursing service irrespective of gender. - gender doesn't dictate the capacity to excel in nursing. - I found that nurses, regardless of gender, were often more attuned to patients' needs and concerns. - gender does not determine the quality of care. - It has reshaped my expectations to focus more on individual qualities rather than gender. - competence and communication are important irrespective of gender - practice should not be tied to gender - It's about the individual and their dedication to patient care. - quality of care isn't tied to gender - qualities like compassion, competence, and communication skills are not bound by gender. - I found that their gender didn't play a significant role in the quality of care - I focus more on the individual nurse's skills and communication rather than their gender. - It's highlighted that each nurse, regardless of gender, has skills and qualities to the profession - where the emphasis is more on the individual's skills, rather than their gender | - one individual's behavior doesn't represent an entire gender or profession - think the hospital need to have both male and female nurses from different parts of Tanzania to bring in different tastes. - both male and female in the nursing profession is an asset. | The need for diversity in nursing | Perception towards gender diversity |
|  | - caring and good communication, are not limited by gender. It's about finding nurses professional - the need for a providing good nursing service irrespective of gender. - gender doesn't dictate the capacity to excel in nursing. - I found that nurses, regardless of gender, were often more attuned to patients' needs and concerns. - gender does not determine the quality of care. - It has reshaped my expectations to focus more on individual qualities rather than gender. - competence and communication are important irrespective of gender - practice should not be tied to gender - It's about the individual and their dedication to patient care. - quality of care isn't tied to gender - qualities like compassion, competence, and communication skills are not bound by gender. - I found that their gender didn't play a significant role in the quality of care - I focus more on the individual nurse's skills and communication rather than their gender. - It's highlighted that each nurse, regardless of gender, has skills and qualities to the profession - where the emphasis is more on the individual's skills, rather than their gender | The nurse’s competency is not bound by gender |  |
| - It made me consider whether I felt more comfortable or understood when the nurse was of a certain gender - I would unconsciously seek out a female nurse when going to the hospital before opting to the available person to attend to me. - I tend to feel more at ease with female nurses. - I would select a female nurse - conservative background, I always feel more comfortable with female nurses for certain personal discussions. - I will always feel more comfortable with female nurses for certain personal discussions. - during discussions about my more personal aspects of my well-being, I somehow felt more comfortable talking to female nurses. - I've often preferred a nurse of the same gender. - if given an opportunity to choose, ill choose a female nurse - I've sometimes felt more comfortable with male nurses during certain personal care situations. - I tend to seek out female nurses if I am in the hospital because I believe they are sympathetic - I have never had a conscious preference for a nurse of a specific gender - I did and do have a preference - I haven't had a specific preference - emotional support. - presence with a sense of security. - women were often associated with caregiving, so having a female nurse made the situation feel less clinical and more comforting. - I believe majority of them possess that sense of peacefulness - been about who is around to help me. - I believe they are sympathetic - I assumed she might understand the emotional aspect better. - I felt more at ease having a female nurse during the recovery process. - I acknowledge the skill of nurses regardless of gender. - For me, it's more about the nurse's on duty and specifically how they communicate and care. - It's about feeling comfortable and confident in the care I receive more than their gender. - My priority is always the nurse's ability to effectively communicate, understand my needs, and provide care - What matters most is the quality of care and the nurse's communication skills. - I look for qualities such as kindness, attentiveness, and professionalism. - the most important thing is that the nurse is competent and caring, regardless of their gender. - they might be more open to these discussions. - been about the individual's competence, communication skills, and ability to connect with patients. - it's about finding a nurse, regardlessof gender, who listens attentively and is available to attend to me. - I've realized it's more about the individual nurse's approach than their gender. - it's always been about the nurse's competence and how comfortable they make me feel. - It was about the individual nurse's ability to make me feel comfortable and confident in their care. Gender wasn't a determining factor - but I also acknowledge that professionalism and competence matter more - It's about the quality of care and who is available to attend to me. - I believed a female nurse might better understand certain aspects of my health concerns. - It's about feeling understood on a deeper level. I assumed a female nurse might sympathize more with the emotional aspects of my situation. - for certain personal discussions. - certain personal discussions. - if I have any sensitive issue to discuss. - personal health issue - during a particularly sensitive situation - sensitive health issue - I felt more comfortable with a female nurse during a sensitive procedure. - cultural thing - I have found myself leaning towards female nurses because I grew up discussing health matters primarily with women in my family. - I have grown up in a home where discussing personal matters with someone of the opposite gender can be uncomfortable | - It made me consider whether I felt more comfortable or understood when the nurse was of a certain gender | Having preference of nurse of specific gender | Preferences of nurse’s gender, reasons, and opinion towards gender preferences |
|  | - I would unconsciously seek out a female nurse when going to the hospital before opting to the available person to attend to me. - I tend to feel more at ease with female nurses. - I would select a female nurse - conservative background, I always feel more comfortable with female nurses for certain personal discussions. - I will always feel more comfortable with female nurses for certain personal discussions. | Having preference of nurse of opposite gender |  |
|  | - during discussions about my more personal aspects of my well-being, I somehow felt more comfortable talking to female nurses. - I've often preferred a nurse of the same gender. - if given an opportunity to choose, ill choose a female nurse - I've sometimes felt more comfortable with male nurses during certain personal care situations. - I tend to seek out female nurses if I am in the hospital because I believe they are sympathetic | Having preference of nurse of same gender |  |
|  | - I have never had a conscious preference for a nurse of a specific gender - I did and do have a preference - I haven't had a specific preference | Having no gender preference for nurses |  |
|  | - emotional support. - presence with a sense of security. - women were often associated with caregiving, so having a female nurse made the situation feel less clinical and more comforting. - I believe majority of them possess that sense of peacefulness - been about who is around to help me. - I believe they are sympathetic - I assumed she might understand the emotional aspect better. - I felt more at ease having a female nurse during the recovery process. | Gender preferences due kind of needed service |  |
|  | - I acknowledge the skill of nurses regardless of gender. - For me, it's more about the nurse's on duty and specifically how they communicate and care. - It's about feeling comfortable and confident in the care I receive more than their gender. - My priority is always the nurse's ability to effectively communicate, understand my needs, and provide care - What matters most is the quality of care and the nurse's communication skills. - I look for qualities such as kindness, attentiveness, and professionalism. - the most important thing is that the nurse is competent and caring, regardless of their gender. - they might be more open to these discussions. - been about the individual's competence, communication skills, and ability to connect with patients. - it's about finding a nurse, regardless of gender, who listens attentively and is available to attend to me. - I've realized it's more about the individual nurse's approach than their gender. - it's always been about the nurse's competence and how comfortable they make me feel. - It was about the individual nurse's ability to make me feel comfortable and confident in their care. Gender wasn't a determining factor - but I also acknowledge that professionalism and competence matter more - It's about the quality of care and who is available to attend to me. - I believed a female nurse might better understand certain aspects of my health concerns. - It's about feeling understood on a deeper level. I assumed a female nurse might sympathize more with the emotional aspects of my situation. | Gender preferences due to nurse’s competency |  |
|  | - for certain personal discussions. - certain personal discussions. - if I have any sensitive issue to discuss. - personal health issue - during a particularly sensitive situation - sensitive health issue - I felt more comfortable with a female nurse during a sensitive procedure. | Gender preferences due to personal sensitive situation |  |
|  | - cultural thing - I have found myself leaning towards female nurses because I grew up discussing health matters primarily with women in my family. - I have grown up in a home where discussing personal matters with someone of the opposite gender can be uncomfortable | Gender preferences due cultural issues |  |
| - Maybe healthcare providers could ask about our preferences during intake or admit processes, giving us the opportunity to express our comfort zones. - Training for healthcare providers - Open communication is important. - Communication and respect is important - create an environment where patients feel comfortable expressing their preferences. - during the admission process, patients could be asked if they have a gender preference for the nurse to attend to them, and efforts can be made to accommodate that preference. - If patients are informed that they have the right to express gender preferences and that the healthcare team will do their best to accommodate, it could lead to more open discussions. - believe open communication could contribute to addressing and directing patient preferences related to gender. - Perhaps asking patients about their preferences in a respectful way could be an option without making assumptions. - The healthcare providers should consider giving us the choice to specify our gender preferences for care, if possible without being pointed out as being bad. - Education both us as patients and the healthcare providers to understanding that good care is not determined by gender, providers - create an environment where we feel comfortable expressing our preferences without judgment is important. - I believe the healthcare providers can communicate and provide education clearly emphasizing that all staff, regardless of gender, are trained to provide high-quality care. This will help build trust with us. | - Maybe healthcare providers could ask about our preferences during intake or admit processes, giving us the opportunity to express our comfort zones. - Training for healthcare providers - Open communication is important. - Communication and respect is important - create an environment where patients feel comfortable expressing their preferences. - during the admission process, patients could be asked if they have a gender preference for the nurse to attend to them, and efforts can be made to accommodate that preference. - If patients are informed that they have the right to express gender preferences and that the healthcare team will do their best to accommodate, it could lead to more open discussions. - believe open communication could contribute to addressing and directing patient preferences related to gender. - Perhaps asking patients about their preferences in a respectful way could be an option without making assumptions. - The healthcare providers should consider giving us the choice to specify our gender preferences for care, if possible without being pointed out as being bad. - Education both us as patients and the healthcare providers to understanding that good care is not determined by gender, providers - create an environment where we feel comfortable expressing our preferences without judgment is important. - I believe the healthcare providers can communicate and provide education clearly emphasizing that all staff, regardless of gender, are trained to provide high-quality care. This will help build trust with us. | Opinion about gender preferences |  |
